# Alma mat(t)er(s): Determinants of early career success in economics

**DOI:** 10.1371/journal.pone.0278320

**Published:** 2022-12-02

**Authors:** Sergey V. Popov

**Affiliations:** Cardiff Business School, Cardiff University, Cardiff, Wales, United Kingdom; Flinders University, AUSTRALIA

## Abstract

We study 6000 author-publication observations to investigate predictors of early career success in six fields of economics. Concentrating on top researchers enables us to control for the effects of ability and effort, and focusing on the start of their careers minimizes distortions from reputation feedback. Our results reveal that the most important predictor for early career success is the ranking of an author’s PhD granting institution, followed by his first placement. Our insights suggest that a counterfactual decrease in the Alma mater of a high ability author, who graduated from a top 10 university, by as little as 10 to 20 ranks, reduces his probability of getting a top 5 publication significantly by 13 percentage points. Lowering the ranking of his Alma mater by another 80 ranks decreases his chances of getting a top publication by a factor of three. Our findings suggest that the economics publication market values Alma mater signals, discounting newcomers graduating from- or working at lower ranked departments.

## Introduction

Since current economic research is used by practitioners such as policy makers with the intention to improve livelihoods of people worldwide, it is important to understand the factors which shape the development of intellectual influence in Economics. Dissemination of scientific results via academic journal publications is key to evaluate the importance of ideas. Top economics journals reach large audiences within and outside the area of economics, and are therefore likely to affect policy making and other relevant decision making processes.

This paper investigates predictors of publications in economics journals and initial job placements in six fields of research: microeconomics, macroeconomics, econometrics, game theory, labor economics and experimental economics. Within each of those branches we study the determinants for acceptance of the *first* ten publications of the *current* top-100 authors ranked on IDEAS as of August 2014. We focus on the top-100 authors in each field to control for heterogeneous abilities: Based on ex-post productivity levels, these authors are arguably capable enough to produce high quality economic research, whose appearance in economics journals is neither because of the prominence of co-authors nor a random fluke. Minimizing the effect of ability is important because academic ability clearly affects every measure of academic success (such as publications, placements, citations, etc). We analyze the first ten publications of each author to minimize the effects of the past and most recent publication history on new publication chances, reputation and the *current* ranking of an author.

We argue that by focusing on ex-post revealed performance rankings we can mitigate the ability heterogeneity in our analysis and disentangle the effects of ability from other factors contributing to early-career publication success. To obtain additional control variables we screen the underlying 600 resumes of our top authors from the fields mentioned above to gain, among other things, information on the author’s first ten publications, their Alma mater, their years of graduation, their gender and the number of co-authors for each of their papers. [1] also investigate data on top economists. They analyze predictors of promotions and labor market mobility over a much longer time horizon (30 years). In contrast to their paper we focus on the very early career of our authors, to correct for reputation concerns. Our main focus is to identify the effects of the ranking of PhD granting institutions on the ranking of publications, controlling for ability. Hence we aim to identify the pure signaling effect associated with the prestige, or a lack thereof, of an author’s Alma mater.

The main insight from our around 6000 author-publication observations is that the ranking of the PhD granting institution, for each author, is a strong predictor for early career publications in higher ranked journals across the six fields mentioned above. Given the specific composition of authors in our data, whose ability levels are arguably high enough to assume away inability as a factor in rejection decisions, we observe that an increase in the ranking of an author’s Alma mater by 100 places, can increase his likelihood to publish in one of the top five journals in economics (*Quarterly Journal of Economics, American Economic Review, Econometrica, Journal of Political Economy, Review of Economic Studies*) by a factor of three. More specifically, consider an author whose probability of having a top 5 publication among equally skilled authors is around 30% and whose Alma mater belonged to the top 10 of all schools worldwide around the time of graduation –which is not uncommon in our sample. If, counterfactually, his Alma mater would have been ranked between 21-30 (100+), his conditional acceptance probability to a top journal would drop significantly to 17% (11.6%), controlling for other factors which may contribute to early career success.

We further observe that the probability to publish in the *Quarterly Journal of Economics* (QJE hereafter) or the *Journal of Political Economy* (JPE hereafter) increases significantly by 4-6 percentage points if an author’s Alma mater has been Harvard or the University of Chicago, even after controlling for the ranking of an author’s PhD granting university—which still remains a highly significant predictor. More specifically, a degree from Chicago increases the probability of publishing in the JPE by about 4 percentage points, whereas a degree of Harvard has no significant effect on publishing in the very same journal. However, a Harvard degree increases the probability of publishing in the QJE by about 6 percentage points, whereas a Chicago degree has no effect on publishing in the QJE. These results are consistent with [2] who show that editors are generally more likely to admit their colleagues’ work to their own journals.

We show that our findings turn out to be very robust against various estimation methods and hold for various subsets of authors. Even after controlling for the current rankings of authors in our regressions, the Alma mater effect remains highly significant across fields. The latter insight further confirms that it is not only skill or innate ability, which predicts early career success in economics. Moreover, our analysis also takes learning considerations into account by controlling for time trends (learning over time), by controlling for accumulated top publications (learning by doing) and by controlling for initial placements (learning from peers). However, even after controlling for these various forms of learning, the ranking of an author’s Alma mater remains a highly significant predictor for early career success in economics.

Furthermore, the Alma mater effect extends to more general and finer journal-rankings. Over the six fields mentioned above, the ranking of the PhD granting institution is the strongest –and in most cases the only– predictor for the ranking of journals in which authors publish early in their careers, controlling for individual ability levels.

Our observations imply that the academic publication market in economics values the Alma mater signal when it comes to publication decisions, which might not be efficient, even after having a significant history of publications. On additional results regarding the inefficiencies in the academic publication market and favoritism, see [3–6]. Intuitively, since authors in our pool are skilled enough to produce high quality research, there should be no significant correlation between the ranking of an author’s Alma mater and the ranking of the journal in which our authors published, even early in their careers. However, in contrast to this intuition, rankings of PhD granting institutions play a major role in the publication success of economists, even after controlling for fields, initial job placements and individual fixed effects. Graduating from a marginally lower ranked Alma mater can easily halve the chances for a top-5 publication, which leaves to wonder whether the Alma mater signal is overvalued or the referees’ signal is uninformative (see [7] on the informativeness of referees’ signals).

Our data on field-specific publications reveal other interesting systematic patterns. First, top macroeconomists tend to publish significantly better than top researchers from most of the other fields considered in this paper. Second, there are strong cohort effects in various sub-fields of economics, which affect publication success ([8] finds that publication chances fell, too, and explains it with the increase of revision rounds requested): it might have been slightly easier to publish in higher ranked journals in certain subfields of economics some decades ago. Third, we observe only mild field-specific gender biases, which are most likely driven by the underrepresentation of females in the top-100 IDEAS rankings. Lastly, our results do not suggest that the number of co-authors per paper has an impact on the ranking of publications of young and high-ability authors, even though co-authorship increased substantially in previous years [9, 10].

We verify our intuition regarding publications using our placement data. First, there are field-specific differences in the median quality of placements. Macroeconomists tend to be the most successful job market candidates followed by microeconomists and econometricians. However, these unconditional differences in rankings of initial placements vanish after controlling for the ranking of the corresponding PhD granting institutions. Our results indicate that each additional rank of an author’s Alma mater translates into a one to one increase in the ranking of his first placement. We also observe that the effects of rankings of *Almae matres* on initial job placements are most important in the fields of labor- and macroeconomics and are significantly less important in game theory and experimental economics. Our results further suggest that gender is not an important explanatory variable for the quality of initial placements. However, this latter insight should be considered with a grain of salt, given that the overwhelming majority of our authors tends to be male.

This paper is organized as follows: we present our data and estimation approach. Then we presents our results, starting with our findings on publication success and followed by our insights on initial placements. Finally, we provide a discussion of our findings, after which we conclude the paper.

## Data and methodology

### Methodology

#### Author pool

Our main intention is to investigate the effects of rankings of PhD granting universities on our relevant left hand side variables. Thus, we essentially aim to estimate the parameters of some “relative” of a standard Mincer equation, with rankings of publications and initial job placements on the left hand side of our regression equations. However, since individual ability and other variables which correlate with schooling are unobservable, we cannot simply use an arbitrary pool of authors in our analysis. Finding exogenous characteristics of ability, which may affect acceptance to certain universities to pursue an academic career is not straightforward and can easily be subject to justified criticisms.

We therefore decided to focus on a pool of authors with *high enough* ability levels for which unobservables are, arguably, negligible and maybe even fairly “homogenous”. Specifically, we focus on the top-100 authors ranked on IDEAS, as of August 2014, (available at http://ideas.repec.org/top/old/1408/; the ranking methodology is explained in [11].) from six relevant fields of economics: microeconomics, macroeconomics, econometrics, game theory, labor economics and experimental economics. Because of overlaps, we therefore consider somewhat less than 600 economists who have influenced the profession along various dimensions. If we approximate the number of economics departments worldwide with a conservative estimate of 500 and further approximate the size of each faculty by 20 people, we might argue that this particular pool of authors represents about top 6% of active economists. Every year, Jobs Openings for Economists, AEA’s job market board, features about 1000 positions. Therefore, in the profession, our almost 600 researchers are a small subpopulation in the right tail of productivity. Given that these authors all had a substantial influence on their corresponding fields ex-post, in the merit-based world, decisions about these authors’ placements and publications should not exhibit systematic patterns regarding arguably idiosyncratic factors like the ranking of *Almae matres* or the first placement ranking.

Our particular selection of authors does not come without costs. While the advantage is that it minimizes complications associated with unobservable ability characteristics, the disadvantage is a plausible lack of external validity and generalizability of our findings. While we might not clearly state that our results hold for every homogeneous pool of authors, we find it unlikely that any shortcoming that we find in this limited setting goes away for the general population of authors.

#### Publications

We study the first 10 publications of our authors, which frequently fall into the pre-tenure period of the authors, when the stakes for publications are high. There are several reasons why we do not consider all publications. First, late career publications of star scientists could be facilitated by the very same current star status. Second, as authors become more experienced over time, they might simply learn how to frame and write papers to increase acceptance probabilities in specific journals or by specific editors. Finally, these star scientists can become editors themselves later in their careers, “turning the tables” and changing underlying motivations [12].

We focus on the early careers of our top economists to lower the chance that these distortions play a role in the publication success of our authors. To verify that early career publications are not affected by their future stardom, we will show below that the current ranking of authors within the top-100 is indeed uncorrelated with the very early career performance of our authors.

Probably one of the most important aspect we control for, by focusing on early career publications, is the reputation concern. Economists standing at the beginning of their careers have very little reputation capital—their affiliations are among the very few observables the relevant decision makers observe. This might be a reason why editors and referees value the affiliation as a signal for ability. However, given that *all* our authors display ex-post high ability levels, there should be little correlation between the ranking of the PhD granting university and the ranking of journals in which they published early in their careers—at least in a pure meritocratic world.

#### Journal rankings

Since we consider the IDEAS ranking of authors, we also consider the IDEAS ranking of journals, which covers more than one thousand different journals across the fields of economics. The ranking is based on several individual ranking methods, taking the harmonic mean and dropping the best and the worst individual ranking for a journal. We provide details and summary statistics on the rankings of publications for our authors, conditional on field, below. By considering various rankings, taking a harmonic mean and correcting for the worst and best ranking, the IDEAS ranking of journals addresses the issues associated with choosing one ranking over another raised in [13]. Besides regressions based on journal rankings, we also study the chances of publishing in top-5 journals: *The Quarterly Journal of Economics, American Economic Review, Econometrica, Journal of Political Economy* and *The Review of Economic Studies*.

#### University rankings

Since we want to establish the effects of rankings of PhD granting schools on early career publication success, we had to find a ranking of universities which reflects the ranks of these institution at around the time at which our authors went through the early stages of their careers. While our impression is that the *current* IDEAS journal-ranking indeed reflects the ranking of journals back in the relevant time for our highly able authors, a similar assessment cannot be given for the current IDEAS university-rankings. Thus, we looked for another economics-department ranking, which is based on information from the 1990’s, covers a wide range of schools and is not only based on US institutions. Indeed, we found a carefully designed ranking for economics departments, based on the years 1993-2003, which ranks 321 economics departments worldwide and uses the methodology of [14]. The ranking can be accessed here: http://econphd.econwiki.com/rank/rallec.htm. Hence, the ranking methodology we use is similar to the one used by [15].

Most of the universities we were looking for could be found on this ranking. Details on the methodology associated with compiling this ranking can be found online on the same website mentioned above, or by request from the author. Whenever a PhD granting institution was not present on this ranking, we assigned a rank of 325. We further generated a dummy variable which takes a value of one whenever an author initially placed at a non-university research institution such as the IMF, the World Bank or a Central Bank.

After having established our basic methodology, we next screened the online resumes of our authors for additional characteristics such as the the years of their graduation, gender, and other variables. In total, out of 600 potential authors contributing to the six fields under concern, 518 unique authors were identified—some belong to the top-100 authors of more than one discipline. The next section provides a more detailed and descriptive analysis of our data.

### Data

Table 1 provides descriptive statistics of our data. The numbers of observations vary from variable to variable due to availability, but there are relatively few missing data points. We provide comparisons of the unconditional distributions of our variables in this section, and move to a multivariate analysis in later sections.

**Table 1:** Descriptive statistics.

	Microeconomics		Macroeconomics		Econometrics	
*PhD year*	1980	*N* = 96	1980	*N* = 97	1983	*N* = 99
	[1944, 1998]		[1955, 2005]		[1956, 2000]	
	(10.9)		(9.4)		(9.6)	
*PhD rank*	40.57	*N* = 100	23.80	*N* = 99	43.21	*N* = 100
	[1, 325]		[1, 325]		[1, 325]	
	(78.27)		(49.36)		(61.06)	
*Placement*	67.70	*N* = 93	107.20	*N* = 94	64.55	*N* = 96
	[1, 325]		[1, 325]		[1, 325]	
	(102.17)		(135.21)		(86.67)	
*Publication Ranks*	0.07	*N* = 1000	0.10	*N* = 1000	0.05	*N* = 1000
	[0, 1]		[0, 1]		[0, 1]	
	(0.20)		(0.24)		(0.13)	
*Publication Year*	1984	*N* = 999	1984	*N* = 999	1986	*N* = 998
	[1949, 2008]		[1958, 2007]		[1952, 2013]	
	(10.50)		(9.89)		(9.99)	
*Had Top 5*	0.84	*N* = 100	0.83	*N* = 100	0.66	*N* = 100
	[0, 1]		[0, 1]		[0, 1]	
	(0.37)		(0.38)		(0.47)	
*Co-authors*	0.6	*N* = 1000	0.5	*N* = 994	0.6	*N* = 1000
	[0, 3]		[0, 4]		[0, 5]	
	(0.73)		(0.66)		(0.70)	
*Non-Ac. Placement*	0.01		0.15		0.01	
*Gender*	0.97		0.95		0.96	
	Game Theory		Labor Economics		Experimental	
*PhD year*	1985	*N* = 97	1982	*N* = 97	1988	*N* = 100
	[1953, 2009]		[1965, 2001]		[1955, 2009]	
	(11.29)		(9.5)		(11.86)	
*PhD rank*	61.43	*N* = 99	37.38	*N* = 99	75.93	*N* = 100
	[1, 325]		[1, 325]		[1, 325]	
	(90.10)		(69.24)		(89.87)	
*Placement*	93.39	*N* = 95	111.88	*N* = 97	114.06	*N* = 95
	[1, 325]		[1, 325]		[1, 325]	
	(113.64)		(128.96)		(116.95)	
*Publication Ranks*	0.07	*N* = 1000	0.12	*N* = 1000	0.07	*N* = 1000
	[0, 1]		[0, 1]		[0, 1]	
	(0.15)		(0.26)		(0.20)	
*Publication Year*	1988	*N* = 999	1986	*N* = 1000	1992	*N* = 1000
	[1949, 2010]		[1961, 2014]		[1957, 2012]	
	(11.85)		(9.47)		(11.84)	
*Had Top 5*	0.71	*N* = 100	0.77	*N* = 100	0.56	*N* = 100
	[0, 1]		[0, 1]		[0, 1]	
*Co-authors*	(0.46)		(0.42)		(0.50)	
	0.6	*N* = 1000	0.6	*N* = 994	0.96	*N* = 1000
	[0, 4]		[0, 10]		[0, 6]	
	(0.86)		(0.75)		(0.92)	
*Non-Ac. Placement*	0		0.07		0	
*Gender*	0.96		0.92		0.87	

Each variable is represented via the average value (rounded for years), the range of the variable (minimum and maximum) contained in [.] and standard errors contained in (.). *N* = the corresponding number of observations.

The first lines in the upper and lower panels of our Table show the average years of graduation of our authors, conditional on field. The range (minimum and maximum) and the standard deviations of the graduation years are illustrated in the lines underneath the average. Testing for differences in the median graduation years, using standard quantile regressions and bootstrapped standard errors across fields of research, reveals that there are no significant differences in graduation years between the fields of microeconomics, macroeconomics, econometrics and labor economics. We essentially run quantile regressions with the individual graduation years on the left hand side against dummy variables for the six fields of research, considering microeconomics as a benchmark. We used F-tests to compare the differences across fields. We double checked our findings using non-parametric rank sum tests to compare the distributions on a bivariate basis. In every case our initial conclusion based on our simple quantile regressions was confirmed. Our top game theorists graduated significantly later than the micro- and macroeconomists (*p*-values are 0.048 and 0.018 respectively) by about 5 to 6 years, but not significantly earlier or later than researchers from the other fields at a 10% level. Similarly, since experimental economics is the youngest among the six fields considered, we observe that the median graduation year among experimental economists is significantly higher than the graduation years of economists in other fields (except), ranging from seven (labor economics, *p*-value 0.006) to ten (microeconomics, *p* − *value* < 0.001).

**Fig 1:**
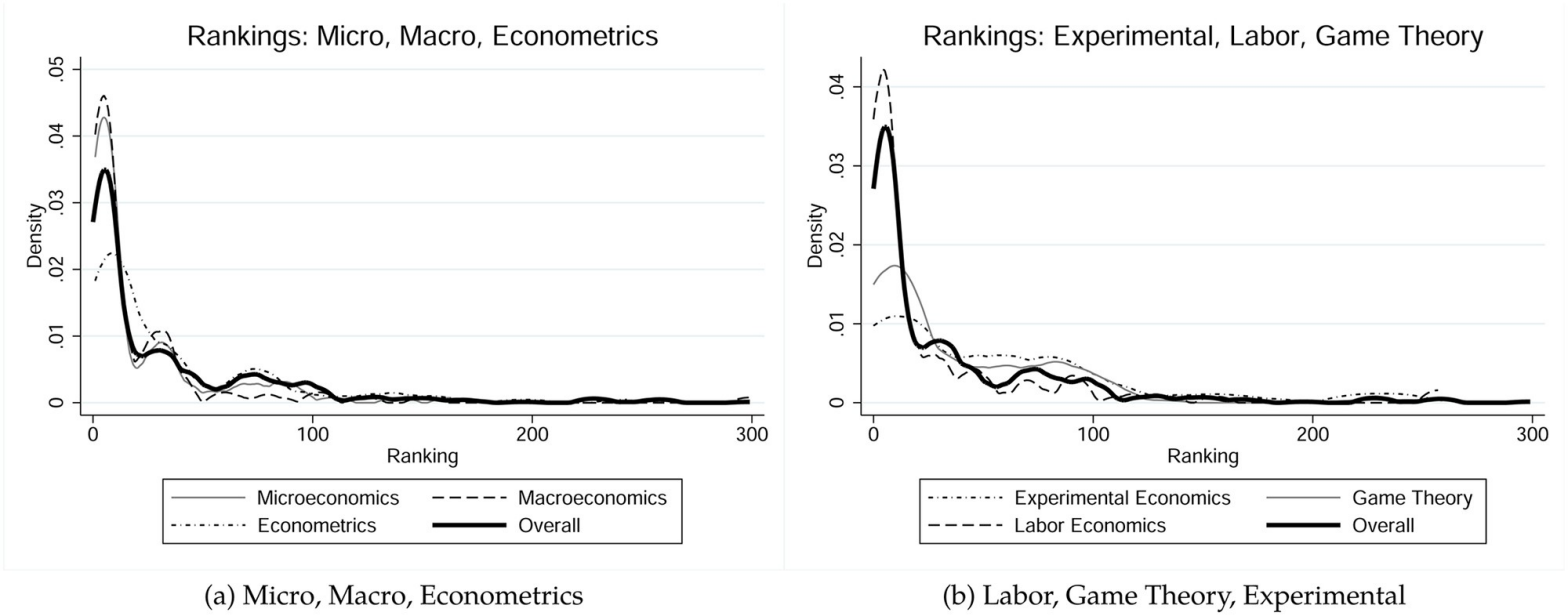
Alma mater distributions, conditional on field. Panel (a) represents estimates for Microeconomics, Macroeconomics, and Econometrics; Panel (b) represents estimates for Experimental Economics, Game Theory, and Labor Economics.

The next lines in our panel show the average, range and standard deviations of the ranks of PhD granting institutions of our authors, conditional on field. We illustrate kernel density estimates of the Alma mater distributions in Fig 1. The overall average ranking of our author’s Alma mater is 47.1 but we observe substantial variation of this measure conditional on field. macroeconomists tend to come from the most prestigious schools with an average ranking of 23.8, followed by labor economists (37.38), microeconomist (40.57), econometricians (43.21), game theorists (61.43) and experimental economists (75.93). At a 10% level there are no significant differences in the average rankings of *Almae matres* among micro-, macro- and labor economists and econometricians.

Game theorists tend to come from significantly lower ranked PhD granting institutions (5% level) if compared to any of the previous four fields. A similar insight holds for experimental economists but there is no significant difference between the rankings of *Almae matres* of experimental economists and game theorists (*p*-value is 0.176). Similar results are obtained if we consider the median instead of the average rankings and test for differences.

**Fig 2:**
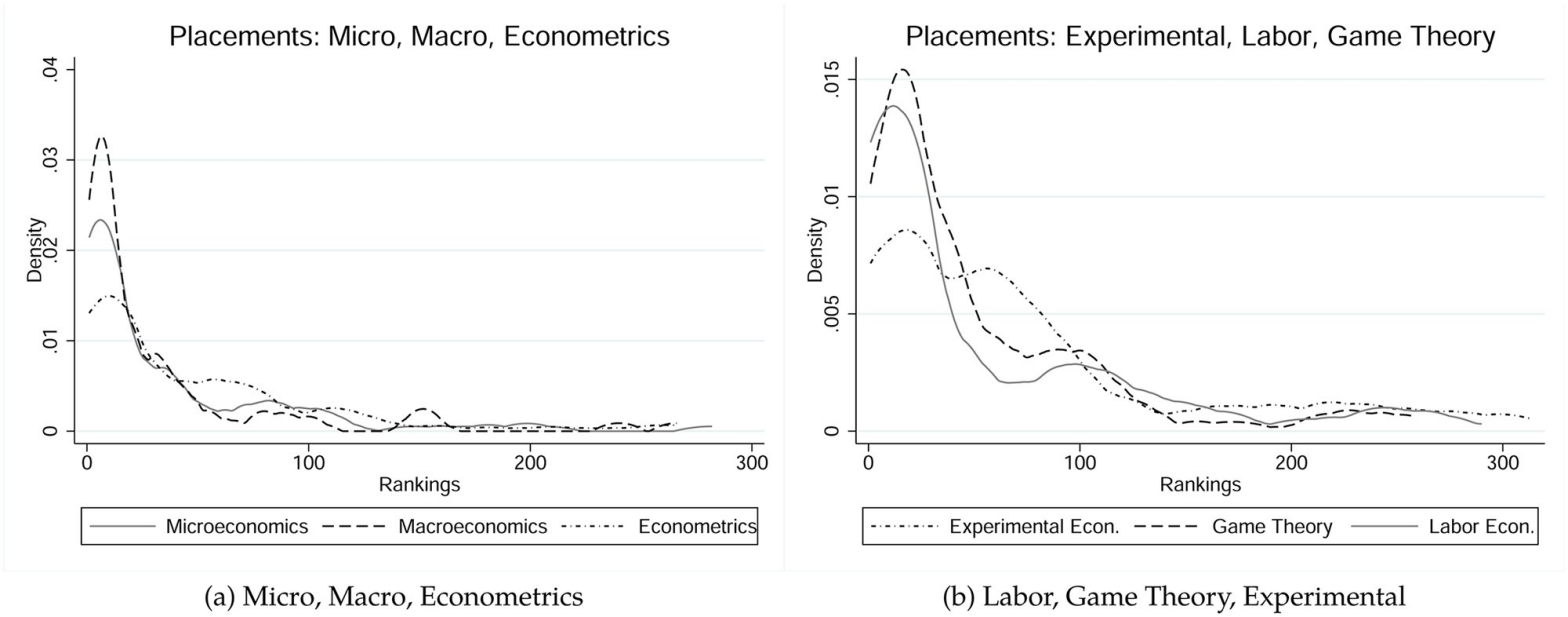
Initial placements distributions, conditional on field. Panel (a) represents estimates for Microeconomics, Macroeconomics, and Econometrics; Panel (b) represents estimates for Experimental Economics, Game Theory, and Labor Economics.

Rankings of initial job placements also vary substantially across fields from 64.5 (econometrics) to 114.06 (experimental). Fig 2 shows kernel density estimates of the distributions of rankings of initial job placements. However, while the average rankings of initial job placements differ quite substantially, the medians reveal that there are no significant differences in initial job placements among five of the six fields. Only the placements of experimental economists differ positively and significantly from all the other fields at a 1% level. Another interesting descriptive feature in our data is the relationship between the rankings of *Almae matres* and the rankings of initial placements. On average, our authors initially went down the ladder by 33.65 ranks, if we ignore non-university placements. While there are substantial differences in average initial placement attrition, which ranges from 17.83 (econometrics) to 55.18 (labor economics), ignoring again non-university placements, there are no significant differences across fields at a 10% level. For further analysis of the initial placements about graduate students in economics in general, not focusing on top-researchers, see [16–18].

**Fig 3:**
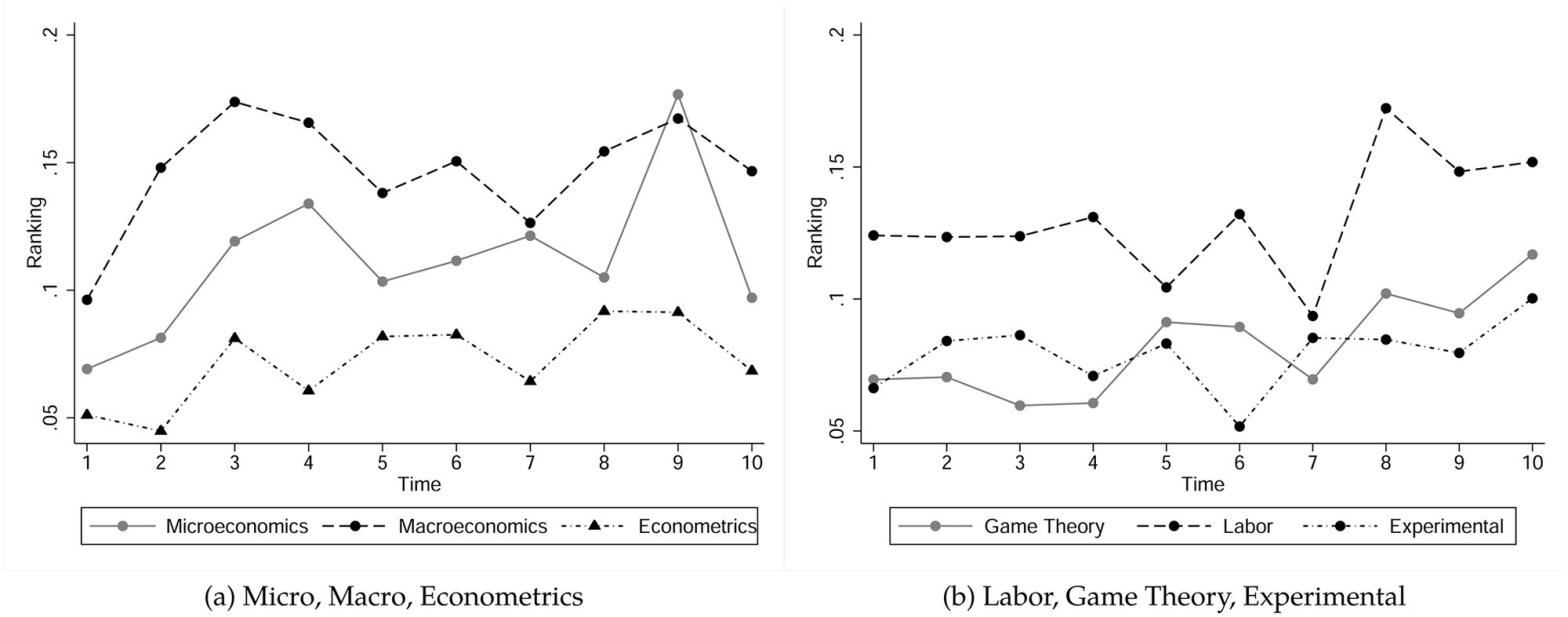
Average rankings of first 10 publications, conditional on field. Panel (a) represents estimates for Microeconomics, Macroeconomics, and Econometrics; Panel (b) represents estimates for Experimental Economics, Game Theory, and Labor Economics.

Next, we describe our data on the rankings of journals in which our top authors published early in their careers. For ease of exposition, we consider the inverse of journal rankings, i.e. a ranking of one corresponds to a publication in the best journal ranked on IDEAS (*Quarterly Journal of Economics*) whereas lower numbers –ranging from zero to one– indicate publications in lower ranked journals. We assigned zeros to journals which are not ranked on IDEAS. We emphasize that none of our statistical results in later sections depend on this particular transformation. Fig 3 displays the average publication rankings of the first ten publications of our authors, conditional on field. The graph reveals that these publication rankings seem to be trending for some fields, which is something we have to take into account later in our analysis. Comparing median journal rankings across fields reveals that macroeconomists are significantly more successful in publishing in higher ranked journals than all other authors at a 1% level. Microeconomists, labor economists and econometricians are equally successful at 10% level and share the second place after macroeconomists. Game theorists are less successful than micro- and macroeconomists at a 1% level, less successful than econometricians and labor economists at a 5% level (*p*−values are 0.04 and 0.012 respectively) and significantly more successful than experimental economists (*p*−value < 0.001).

The median first publication year of our authors is 1987. Regarding the relationship across fields, we obtain the same pattern and ordering as with the years of graduation described above. We skip the presentation of these results for brevity.

Looking at the fraction of top-5 publications across fields (*Quarterly Journal of Economics, American Economic Review, Econometrica, Journal of Political Economy, Review of Economic Studies*), we observe that the fraction of authors with at least one top 5 publication is the largest in microeconomics, macroeconomics and labor economics. Moreover, these three fields do not generate significant differences in those fractions at a 1% level. Authors in the fields of experimental economics and econometrics generate a smaller fraction of authors with at least one top publication than the aforementioned fields at a 5% level. Experimental economists generate the smallest fraction of authors with at least one top publication, if compared to four out of the five remaining fields. Only if we compare econometrics to experimental economics, we obtain that these two fields do not generate a significant difference in this particular fraction (*p*-value is 0.147).

There are only mild differences in the number of authors per paper across fields in our data. Overall, the average number of authors per paper in our pool of researchers is 1.68. Comparing both median and means of the number of co-authors across fields reveals that there are no significant differences across five out of six fields. Only the number of authors per paper in experimental economics exceeds the number of authors in every other field at least at a 5% significance level.

Lastly, we observe that the fraction of initial non-university placements is naturally the largest in macroeconomics and negligible in other fields among our authors. The fraction of male authors is overwhelmingly high in our data and ranges, conditional on field, from 87% (experimental economics) to 97% (microeconomics).

## Results

Next, we turn to our analysis of factors influencing the success of early-career publications and initial job placements in economics within the six fields we consider.

### Publication rankings regressions

In a first step we estimate a standard random effects model, with standard errors clustered on the author level, to predict the inverse rankings of individual publications. As controls we use authors’ *Gender*; authors’ year of graduation (*PhD Year*)—subdivided into several intervals to control for non-linear cohort effects; the number of co-authors per paper (*Co-authors*); sequentiality (*Trend*; ranging from one to ten) to control for experience over time; and the rankings of their *Almae matres* (*PhD Rank*). Note that the inverse journal ranking is decreasing in nature, and ranges from zero to one. We chose this transformation for the ease of presenting our results, however our findings can be verified to hold under other transformations.

#### Field regressions

We initially estimate these random effect models for each field separately. We initially tested whether our left hand side variable shows significant autocorrelation using [19]. We do not observe any significant autocorrelation among the rankings of journals in which our authors publish, both overall and within the corresponding fields. The results from our random effects estimations are shown in Table 2.

**Table 2:** Random effects regressions: Ranking of publications using IDEAS journal rankings.

	(1) Micro	(2) Macro	(3) Econometrics	(4) GameTheory	(5) Labor	(6) Experimental
*Gender*	-0.00247	-0.0427	0.0316	-0.0733**	-0.0733	-0.0192
	(0.0592)	(0.0529)	(0.0306)	(0.0355)	(0.0614)	(0.0290)
*Cohort 1960–1979*	-0.0421**	-0.0669	-0.00545	0.0266	-0.0577***	-0.0212
	(0.0179)	(0.103)	(0.0238)	(0.0460)	(0.0177)	(0.0861)
*Cohort 1980–1999*	-0.0231	-0.0777	-0.00336	0.0165	-0.0481***	-0.0178
	(0.0161)	(0.103)	(0.0235)	(0.0457)	(0.0155)	(0.0859)
*Cohort ≥ 2000*	-0.0533	-0.0416	0.00549	-0.00824	-0.0230	0.0318
	(0.0408)	(0.109)	(0.0226)	(0.0617)	(0.0592)	(0.0971)
*PhD Rank*	-0.000210**	-0.000596***	-0.000237**	-0.000125	-0.000549***	-0.000393***
	(0.0000940)	(0.000131)	(0.000107)	(0.0000852)	(0.000152)	(0.0000910)
*Co-authors*	0.00180	-0.00663	-0.00482	0.0135**	0.00731	-0.000201
	(0.00959)	(0.0139)	(0.00618)	(0.00691)	(0.0105)	(0.00574)
*Trend*	0.00491**	0.00239	0.00347***	0.00378**	0.00337	0.00169
	(0.00220)	(0.00246)	(0.00126)	(0.00160)	(0.00271)	(0.00197)
Observations	960	964	988	978	978	1000
*R* ^2^	0.07	0.10	0.09	0.10	0.17	0.15

Clustering standard errors on an author level. Standard errors in parentheses.

*p < 0.10,

**p < 0.05,

***p < 0.01.

Gender is a dummy variable that takes a value of one for male authors; we include cohort dummies to control for non-linear cohort-effects. Benchmark is the cohort < *1960*. PhD rank is the ranking of an author’s Alma mater. Co-authors contains the number of authors per paper. Trend denotes the paper’s number in the author’s publication history: 1 means first, 3 means third, and so on. *R*^2^’s are overall *R*^2^.

Our results show that the ranking of an author’s Alma mater is a highly significant predictor (1-5% significance level), for the rankings of journals in which our highly skilled authors publish early in their careers in five out of six fields. An increase in an author’s Alma mater ranking (going from better to worse ranked university) decreases the ranking of an author’s publication. Using this simplest estimation method, our results suggest that the ranking of an author’s Alma mater is not a significant explanatory variable in the field of game theory. Instead, we observe that for game theorists sequentiality matters at a 5% significance level, as well as gender and the number of co-authors. We interpret the significance of the sequentiality variable as an indication of the relative importance of experience in publishing articles, or maybe the importance of having a certain amount of contributions to build up reputation. The few female game theorists in our sample also seem to be slightly more successful than their male colleagues, although the latter result is only significant at a 10% level. Co-authorship also matters to explain the ranking of early career publications among game theorists.

Sequentiality, in addition to the ranking of an author’s Alma mater, also matters for econometricians (5% level) and also contributes to early career success of microeconomists. Another interesting feature is that for labor economists, later graduation years matter. The earlier a labor economist graduated, the lower ranked are his publications.

Next, we add to our previous regressions the variable *Initial Placement*, to control for the ranking of the university an was author initially placed. The results are shown in Table 3. We observe that none of our previous results change substantially and that the ranking of initial placements don’t matter for five out of six fields. Only in the field of labor economics, we observe that the ranking of an author’s initial placement matter significantly: The better a labor economist places, the better ranked are his early career publications. However, the ranking of an author’s Alma mater remains a significant contributing factor to early career success.

**Table 3:** Random effects regressions: Controlling for initial placement.

	(1) Micro	(2) Macro	(3) Econometrics	(4) GameTheory	(5) Labor	(6) Experimental
*Gender*	0.0528***	-0.0487	0.0316	-0.0727**	-0.0712	-0.0149
	(0.0183)	(0.0471)	(0.0308)	(0.0317)	(0.0570)	(0.0289)
*Cohort 1960–1979*	-0.0394*	-0.0634	-0.00565	0.0300	-0.0468**	-0.0175
	(0.0202)	(0.103)	(0.0231)	(0.0448)	(0.0185)	(0.0877)
*Cohort 1980–1999*	-0.0289	-0.0636	-0.00106	0.0207	-0.0411***	-0.00986
	(0.0200)	(0.104)	(0.0230)	(0.0445)	(0.0151)	(0.0876)
*Cohort ≥ 2000*	-0.0697**	0.00249	0.0119	0.00572	-0.0189	0.0484
	(0.0312)	(0.104)	(0.0258)	(0.0652)	(0.0901)	(0.0979)
*PhD Rank*	-0.000227*	-0.000538***	-0.000224**	-0.0000793	-0.000373**	-0.000360***
	(0.000130)	(0.000152)	(0.000110)	(0.0000975)	(0.000170)	(0.0000995)
*Co-authors*	0.00886	-0.00481	-0.00445	0.0131*	0.00741	0.000293
	(0.00982)	(0.0138)	(0.00637)	(0.00712)	(0.0105)	(0.00617)
*Trend*	0.00442*	0.00226	0.00357***	0.00397**	0.00396	0.00165
	(0.00231)	(0.00257)	(0.00130)	(0.00166)	(0.00275)	(0.00207)
*Initial Placement*	0.0000641	-0.000118	-0.0000405	-0.0000884	-0.000239***	-0.0000918
	(0.000104)	(0.000107)	(0.0000837)	(0.0000782)	(0.0000762)	(0.0000897)
Observations	930	934	958	948	959	950
*R* ^2^	0.07	0.10	0.09	0.10	0.17	0.15

Clustering standard errors on an author level. Standard errors in parentheses.

*p < 0.10,

**p < 0.05,

***p < 0.01.

Gender is a dummy variable that takes a value of one for male authors; We include cohort dummies instead to control for non-linear cohort-effects. Benchmark is the cohort < *1960*. PhD rank is the ranking of an author’s Alma mater. Co-authors contains the number of authors per paper. Trend denotes the paper’s number in the author’s publication history: 1 means first, 3 means third, and so on. Initial Placement captures the ranking of the university of the author’s initial placement. *R*^2^’s are overall *R*^2^.

Next, we address the issue that our transformed ranking measure is limited from above and below. We again test the relationship between the same variables as above, with the difference that we are now using panel Tobit estimators. The results are shown in Table 4. Our results reveal that the ranking of *Almae matres* is now significant for all the six fields, having the expected negative sign. This suggests that game theorists potentially publish a significant number of papers outside economics, in the fields such as Operations Research or Mathematics, which might simply not be ranked on IDEAS, thereby obtaining a value of zero in our (inverse) rankings. Either way, the variable *PhD Rank* is now significant at a 5% level for all the six fields we consider.

**Table 4:** Panel Tobit regressions: Ranking of publications using IDEAS journal rankings.

	(1) Micro	(2) Macro	(3) Econometrics	(4) GameTheory	(5) Labor	(6) Experimental
*Gender*	-0.00250	-0.0618	0.0833*	-0.0912*	-0.0931*	-0.0409
	(0.0479)	(0.0677)	(0.0427)	(0.0487)	(0.0518)	(0.0419)
*Cohort 1960–1979*	-0.0421	0.00310	0.0507	0.141*	-0.0759	0.0678
	(0.0346)	(0.0885)	(0.0518)	(0.0785)	(0.141)	(0.109)
*Cohort 1980–1999*	-0.0231	-0.0195	0.0779	0.145*	-0.0679	0.0935
	(0.0340)	(0.0892)	(0.0511)	(0.0778)	(0.141)	(0.107)
*Cohort ≥ 2000*	-0.0533	0.0287	0.0819	0.102	-0.0332	0.164
	(0.0521)	(0.119)	(0.0933)	(0.0852)	(0.160)	(0.123)
*PhD Rank*	-0.000210**	-0.00107***	-0.000349***	-0.000249**	-0.000780***	-0.000522***
	(0.000101)	(0.000327)	(0.000133)	(0.000113)	(0.000219)	(0.000160)
*Co-authors*	0.00198	-0.0106	-0.0100	0.0207**	0.0126	-0.00463
	(0.00913)	(0.0156)	(0.00765)	(0.00820)	(0.0150)	(0.0100)
*Trend*	0.00490**	0.00329	0.00475***	0.00616***	0.00658*	0.00383
	(0.00218)	(0.00321)	(0.00169)	(0.00213)	(0.00345)	(0.00278)
Observations	960	964	988	978	978	1000

Standard errors in parentheses.

*p < 0.10,

**p < 0.05,

***p < 0.01.

Lower bound: 0, Upper bound: 1. Gender is a dummy variable that takes a value of one for male authors; We include cohort dummies to control for non-linear cohort-effects. Benchmark is the cohort < *1960*. PhD rank is the ranking of an author’s Alma mater. Co-authors contains the number of authors per paper. Trend denotes the paper’s number in the author’s publication history: 1 means first, 3 means third, and so on.

Controlling for discontinuities on the boundaries of our endogenous variable via our Tobit estimator reveals that sequentiality matters now, at a 5%-10% significance level, in four out of the six fields, and has a positive coefficient. Put differently, the rankings of publications tend to improve over time, except for macroeconomists and experimentalists. Furthermore, we observe that the number of co-authors correlates significantly (5% level) and positively with the ranking of journals in which game theorists publish.

We again control for initial placements in a separate step. The results are shown in Table 5. We again observe that initial placements only matter for labor economists and inclusion of this variable has no substantial effect on the significance of the remaining variables.

**Table 5:** Panel Tobit regressions: Controlling for initial placement.

	(1) Micro	(2) Macro	(3) Econometrics	(4) GameTheory	(5) Labor	(6) Experimental
*Gender*	0.0527	-0.0692	0.0829*	-0.0915*	-0.0906*	-0.0356
	(0.0601)	(0.0690)	(0.0438)	(0.0495)	(0.0481)	(0.0427)
*Cohort 1960–1979*	-0.0393	0.00710	0.0520	0.147*	-0.0647	0.0728
	(0.0345)	(0.0894)	(0.0526)	(0.0799)	(0.132)	(0.110)
*Cohort 1980–1999*	-0.0289	-0.00147	0.0826	0.153*	-0.0638	0.107
	(0.0345)	(0.0906)	(0.0520)	(0.0792)	(0.132)	(0.109)
*Cohort ≥ 2000*	-0.0697	0.0825	0.0959	0.117	-0.0647	0.184
	(0.0582)	(0.133)	(0.0965)	(0.0885)	(0.161)	(0.127)
*PhD Rank*	-0.000227**	-0.000997***	-0.000327**	-0.000159	-0.000486**	-0.000511***
	(0.000115)	(0.000338)	(0.000145)	(0.000133)	(0.000223)	(0.000180)
*Co-authors*	0.00916	-0.00806	-0.00997	0.0205**	0.0137	-0.00641
	(0.00951)	(0.0161)	(0.00791)	(0.00845)	(0.0149)	(0.0107)
*Trend*	0.00441*	0.00278	0.00508***	0.00628***	0.00746**	0.00406
	(0.00227)	(0.00331)	(0.00176)	(0.00221)	(0.00346)	(0.00292)
*Initial Placement*	0.0000642	-0.000148	-0.0000755	-0.000155	-0.000361***	-0.0000804
	(0.0000917)	(0.000119)	(0.000105)	(0.000107)	(0.000110)	(0.000145)
Observations	960	964	988	978	978	1000

Standard errors in parentheses.

*p < 0.10,

**p < 0.05,

***p < 0.01.

Lower bound: 0, Upper bound: 1. Gender is a dummy variable that takes a value of one for male authors; We include cohort dummies to control for non-linear cohort-effects. Benchmark is the cohort < *1960*. PhD rank is the ranking of an author’s Alma mater. Co-authors contains the number of authors per paper. Trend denotes the paper’s number in the author’s publication history: 1 means first, 3 means third, and so on. Initial Placement captures the ranking of the university of the author’s initial placement.

#### Aggregate dataset

Next, we run random effects regressions using all our observations across fields, considering only unique author-observations, and adding the field dummy as a control variable. This left us with 5180 observations. To add the field fixed effects, whenever an author was among the top-100 in more than one field, we randomly assigned the author to one of the fields he contributed to. In a separate step we also dropped the authors who contributed to more than one field an re-ran our regressions. We also used panel Tobit estimators and re-ran our random effects regressions, acknowledging our findings from above. However, none of these changes our results qualitatively.

Table 6 shows our estimation results using all our observations, presenting various nested models. First, we observe that the ranking of the PhD granting institution has the expected sign and is significant for all our models. We observe that the year of graduation (*PhD Year*) and an author’s gender play no role in explaining the ranking of an author’s publications. Sequentiality (*Trend*) has a positive impact, suggesting that the ranking of publications of authors increase over time during the early career. We also control for the ranking of an author’s initial placement, which is also significant and has the same sign as the ranking of the PhD granting institution. Hence, the better the initial placement, the better the early career publications. Interestingly, even after controlling for initial placements, the ranking of the PhD granting institution remains significant, although loses magnitude. The two variables correlate, using a Spearman rank-correlation, significantly and positively. Overall the correlation is below 0.6 and ranges from 0.5-0.68, conditional on field. We also control for the underlying fields, using microeconomics as a benchmark, via field dummies. The results confirm our field-specific analysis from above that macroeconomists remain the most successful early-career economists. We also add interaction terms into our regressions, interacting *Trend* and *PhD Rank* (*Interaction 1*) as well as *Trend* and *Placement* (*Interaction 2*). The interaction terms check whether the initial Alma mater and placement effects vanish as the careers of our authors progress. The results suggest that the initial effects are fairly sticky, since both coefficients are insignificant. Lastly, we also add the variable *Duration* to our regressions, which measures the years between the first and the last of the ten publications of our authors. Duration might therefore be a proxy for early career life-cycle productivity [20], or another proxy for the author’s productivity. This latter variable is insignificant in all our regressions.

**Table 6:** Nested random effects regressions using all unique author observations.

	(1) All1	(2) All2	(3) All3	(4) All4	(5) All5
*PhD Rank*	-0.000402***	-0.000349***	-0.000284***	-0.000272***	-0.000248***
	(0.0000476)	(0.0000486)	(0.0000549)	(0.0000565)	(0.0000668)
*Macro*		0.0363**	0.0412***	0.0363**	0.0384**
		(0.0149)	(0.0156)	(0.0158)	(0.0158)
*Econometrics*		-0.0357***	-0.0351***	-0.0392***	-0.0383***
		(0.0104)	(0.0109)	(0.0115)	(0.0114)
*Game Theory*		-0.0205*	-0.0184	-0.0232*	-0.0219*
		(0.0119)	(0.0125)	(0.0128)	(0.0127)
*Labor*		0.0212	0.0242*	0.0166	0.0169
		(0.0142)	(0.0147)	(0.0144)	(0.0143)
*Experimental*		-0.0206	-0.0142	-0.0236	-0.0216
		(0.0141)	(0.0151)	(0.0162)	(0.0163)
*Initial Placement*			-0.000105**	-0.000107**	-0.000126*
			(0.0000438)	(0.0000450)	(0.0000658)
*Gender*				-0.0301	-0.0279
				(0.0206)	(0.0209)
*PhD Year*				0.000290	0.000341
				(0.000414)	(0.000419)
*Trend*				0.00340***	0.00336**
				(0.000947)	(0.00134)
*Interaction 1*					-0.00000445
					(0.00000945)
*Interaction 2*					0.00000259
					(0.00000933)
*Duration*					0.00174
					(0.00110)
Observations	5150	5150	4900	4840	4840
*R* ^2^	0.095	0.163	0.17	0.17	0.18

Standard errors in parentheses.

*p < 0.10,

**p < 0.05,

***p < 0.01.

Dependent variable: Ranking of publications using IDEAS journal rankings. Gender is a dummy variable that takes a value of one for male authors; All unique authors considere, controlling for field dummies. PhD rank is the ranking of an author’s Alma mater. Co-authors contains the number of authors per paper. Trend denotes the paper’s number in the author’s publication history: 1 means first, 3 means third, and so on. PhD Year is the year of graduation per author. Interaction PhD Ranking is the interaction between Trend and the ranking of an author’s Alma mater. Interaction PhD placement is the interaction between the initial placement of an author and Trend. Placement is the ranking of the initial placement of an author. Duration is the time span between the first and the last of the (first) ten publications of an author.

**Fig 4:**
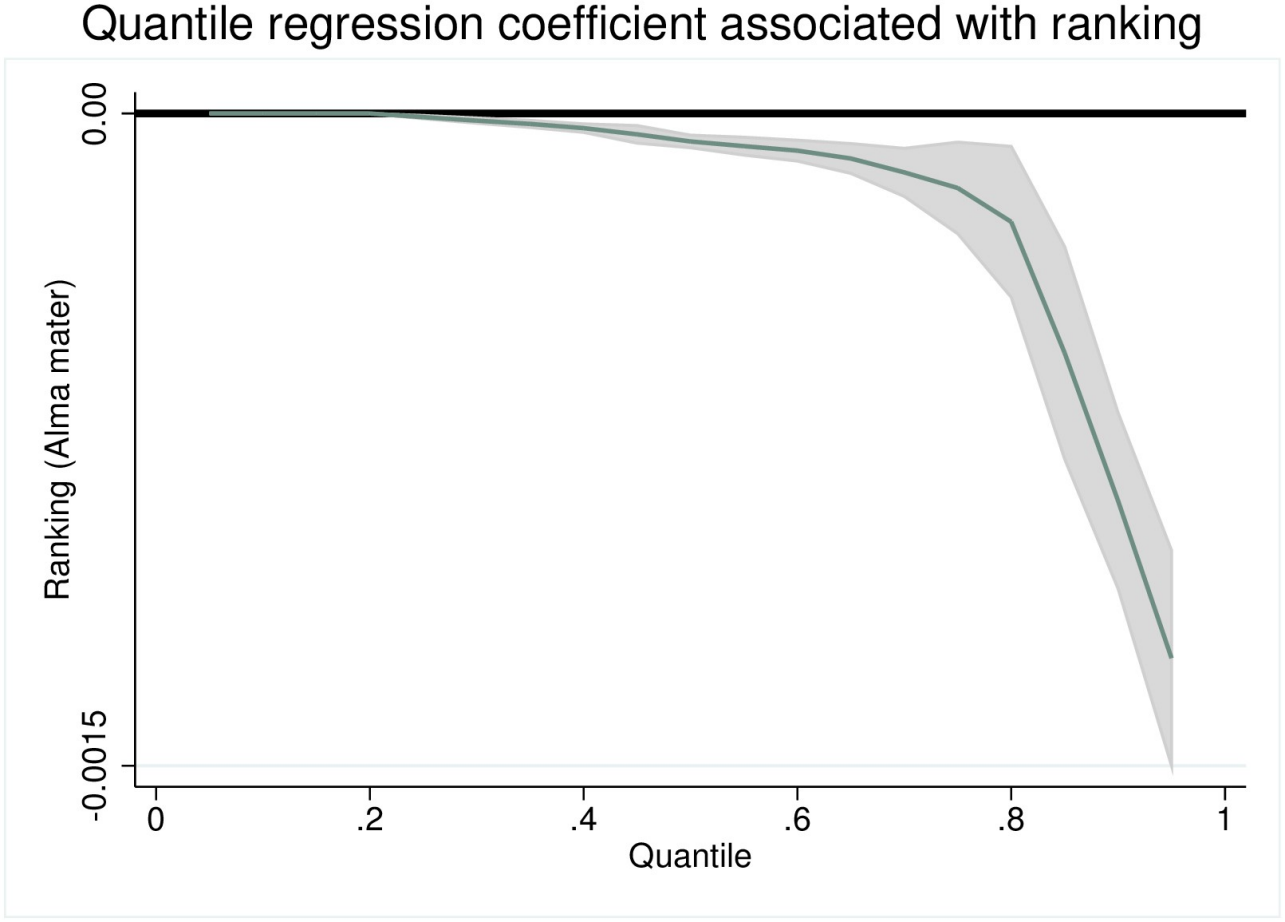
Quantile regression coefficient associated with the effect of rankings of *Almae matres* on journal-publication rankings. Solid line: Zero reference line. 95% confidence intervals in shaded area.

**Fig 5:**
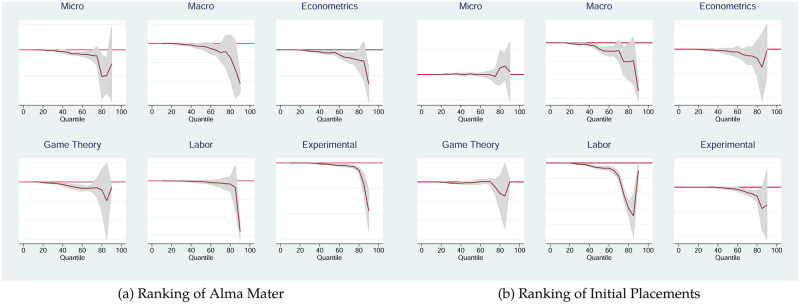
Quantile regression coefficients for controls. From a regression with ranked publications on the left hand side and the same controls as above on the right hand side. The horizontal line indicates the zero-line. The shaded areas indicate 95% confidence bounds. Panel (a) represents the coefficient for the ranking of Alma Mater; Panel (b) represents the coefficient for the ranking of initial placement.

We also estimate the same combined model from above, over all fields, using panel-quantile regression method (see [21] for details). Our observations from our random effects model do not change qualitatively if we consider the effects of our right hand side variables on the conditional quantiles of our left hand side variable above the 20th percentile. More specifically, the ranking of the PhD granting institution has no effect on acceptance to journals ranked below the 20th percentile of our journals and has a significant and negative effect on acceptance to journals belonging to the “top-80%” of *all* journals ranked on IDEAS. Moreover, as the ranking of the journal increases, the effect of the ranking of the PhD granting university becomes significantly stronger. Hence, the Alma mater effect becomes increasingly important towards better ranked journals. We depict the quantile regression coefficient associated with an author’s Alma mater in Fig 4. The coefficients associated with each field are depicted in Fig 5a. The coefficients associated with initial placements are shown in Fig 5b.

We performed other robustness checks to test our results. We used ordered probit regressions, simple OLS with appropriately clustered standard errors, tested various nested models etc. None of these changes in estimation methods change our main result, that the most important predictor for our left hand side variable is indeed the ranking of an author’s Alma mater. For the sake of brevity the presentation of these robustness checks was skipped but can be made available upon request. We also checked if our results change if we only focus on papers published in ranked journals (Table 7) or solo-authored publications (Table 8). Both checks indicate again that the main predictor for early career success is an author’s Alma mater. Although not all robustness checks entered the main body of our paper, we want to present a few additional findings, which might also be considered as additional robustness checks, but are also interesting by themselves.

**Table 7:** Random effects regressions: Ranking of publications omitting unranked publications.

	(1) Micro	(2) Macro	(3) Econometrics	(4) GameTheory	(5) Labor	(6) Experimental
*Gender*	0.0457**	-0.0360	0.00366	-0.0801**	-0.0637	-0.000794
	(0.0195)	(0.0547)	(0.0429)	(0.0387)	(0.0553)	(0.0343)
*Cohort 1960–1979*	-0.0409	-0.187***	-0.245	-0.130***	-0.000837	-0.153***
	(0.0270)	(0.0610)	(0.241)	(0.0138)	(0.0242)	(0.0261)
*Cohort 1980–1999*	-0.0446*	-0.177***	-0.260	-0.154***	0.00380	-0.144***
	(0.0261)	(0.0628)	(0.241)	(0.0116)	(0.0211)	(0.0267)
*Cohort ≥ 2000*	-0.103***	-0.112*	-0.233	-0.162**	0.0311	-0.0754
	(0.0397)	(0.0619)	(0.241)	(0.0677)	(0.121)	(0.0588)
*PhD Rank*	-0.000279**	-0.000784***	-0.000218*	-0.0000837	-0.000497**	-0.000528***
	(0.000138)	(0.000267)	(0.000124)	(0.000109)	(0.000219)	(0.000138)
*Co-Authors*	0.00917	-0.00371	-0.000509	0.0111	0.00539	0.00637
	(0.0110)	(0.0168)	(0.00811)	(0.00841)	(0.0150)	(0.00920)
*Trend*	0.00485*	0.00278	0.00400**	0.00450**	0.00390	0.00147
	(0.00264)	(0.00331)	(0.00161)	(0.00209)	(0.00335)	(0.00294)
*Placement Rank*	0.0000633	-0.000142	-0.0000730	-0.0000752	-0.000248**	-0.000162
	(0.000111)	(0.000119)	(0.000104)	(0.0000997)	(0.0000971)	(0.000120)
Observations	759	778	755	735	745	685
*R* ^2^	0.03	0.09	0.16	0.13	0.13	0.15

Clustering standard errors on an author level. Standard errors in parentheses.

*p < 0.10,

**p < 0.05,

***p < 0.01.

Gender is a dummy variable that takes a value of one for male authors; PhD Year is a vector containing the year of graduation for each author. PhD rank is the ranking of an author’s Alma mater. Co-authors contains the number of authors per paper. Trend denotes the paper’s number in the author’s publication history: 1 means first, 3 means third, and so on. *R*^2^’s are overall *R*^2^.

**Table 8:** Random effects regressions: Ranking of publications using only solo authored papers.

	(1) Micro	(2) Macro	(3) Econometrics	(4) GameTheory	(5) Labor	(6) Experimental
*Gender*	0.0280	-0.103	0.0247	-0.163***	-0.0149	-0.0322
	(0.0388)	(0.0754)	(0.0511)	(0.0397)	(0.0414)	(0.0464)
*Cohort 1960–1979*	-0.0210	-0.0748	-0.0404	0.0420	-0.106***	-0.0267
	(0.0272)	(0.118)	(0.0253)	(0.0319)	(0.0205)	(0.0991)
*Cohort 1980–1999*	-0.0264	-0.0753	-0.0475*	0.0293	-0.0968***	-0.0519
	(0.0278)	(0.119)	(0.0248)	(0.0315)	(0.0218)	(0.0979)
*Cohort ≥ 2000*	-0.0892**	-0.0748	-0.0525	0.0651	0.00183	0.00890
	(0.0359)	(0.129)	(0.0351)	(0.0668)	(0.129)	(0.120)
*PhD Rank*	-0.000322*	-0.000359**	-0.000563***	-0.000147**	-0.000490**	-0.000316***
	(0.000192)	(0.000156)	(0.000170)	(0.0000743)	(0.000249)	(0.0000758)
*Trend*	0.00102	-0.00107	0.00478**	-0.000632	0.00282	0.00186
	(0.00320)	(0.00370)	(0.00213)	(0.00229)	(0.00331)	(0.00304)
*Placement Rank*	0.000172	-0.000254**	-0.0000165	-0.0000887	-0.000224***	-0.000108
	(0.000209)	(0.000118)	(0.000134)	(0.0000799)	(0.0000844)	(0.0000849)
Observations	485	511	474	441	476	340
*R* ^2^	0.03	0.09	0.16	0.13	0.13	0.15

Clustering standard errors on an author level. Standard errors in parentheses.

*p < 0.10,

**p < 0.05,

***p < 0.01.

Gender is a dummy variable that takes a value of one for male authors; PhD Year is a vector containing the year of graduation for each author. PhD rank is the ranking of an author’s Alma mater. Co-authors contains the number of authors per paper. Trend denotes the paper’s number in the author’s publication history: 1 means first, 3 means third, and so on. *R*^2^’s are overall *R*^2^.

#### Deeper incisions

First, we split our sample along the median ranked author for each subfield and re-run our random effect regressions from above for the authors ranked above and below the median. Within each of these subsets variation in ability levels should be even less important, in particular towards the top. The results for the authors above the median are shown in Table 9 (see also Table 10 for those below the median). We observe again that the Alma mater matters in five out of the six fields at least at a 5% level.

**Table 9:** Random effects regressions: Top 50 authors per field.

	(1) MicroTop	(2) MacroTop	(3) EconometricsTop	(4) GameTheoryTop	(5) LaborTop	(6) ExperimentalTop
*Gender*		0.127***	0.0852**	-0.0793*	0.132***	-0.0266
		(0.0461)	(0.0336)	(0.0447)	(0.0358)	(0.0532)
*Cohort 1960–1979*	-0.0539**	-0.0799***	-0.0227*	-0.0342**	-0.0588***	0.00491
	(0.0267)	(0.0227)	(0.0116)	(0.0167)	(0.0227)	(0.0895)
*Cohort 1980–1999*	-0.0401	-0.120***	-0.0237*	-0.0461***	-0.0781***	-0.00355
	(0.0258)	(0.0299)	(0.0130)	(0.0133)	(0.0225)	(0.0929)
*Cohort ≥ 2000*	-0.0852**	-0.0340	-0.0400	-0.114***	0.101	0.209**
	(0.0405)	(0.0368)	(0.0292)	(0.0214)	(0.157)	(0.0947)
*PhD Rank*	-0.000271**	-0.00178***	-0.000621***	-0.0000316	-0.000822**	-0.000386**
	(0.000125)	(0.000678)	(0.000144)	(0.000120)	(0.000366)	(0.000156)
*Co-authors*	0.00601	0.000675	-0.0225**	0.0205*	0.0198	-0.00412
	(0.0123)	(0.0183)	(0.0111)	(0.0106)	(0.0171)	(0.00920)
*Trend*	0.00847**	0.00196	0.00416**	0.00379	0.00281	0.00450*
	(0.00332)	(0.00405)	(0.00208)	(0.00241)	(0.00460)	(0.00263)
*Initial Placement*	0.0000173	0.00000999	0.0000596	-0.0000575	-0.000118	-0.0000450
	(0.000136)	(0.000183)	(0.000146)	(0.000111)	(0.000106)	(0.000146)
Observations	480	499	500	489	499	500
*R* ^2^	0.11	0.17	0.31	0.22	0.27	0.19

Clustering standard errors on an author level. Standard errors in parentheses.

*p < 0.10,

**p < 0.05,

***p < 0.01.

Gender is a dummy variable that takes a value of one for male authors; We include cohort dummies to control for non-linear cohort-effects. Benchmark is the cohort < *1960*. PhD rank is the ranking of an author’s Alma mater. Co-authors contains the number of authors per paper. Trend denotes the paper’s number in the author’s publication history: 1 means first, 3 means third, and so on. Initial Placement captures the ranking of the university of the author’s initial placement. *R*^2^’s are overall *R*^2^.

**Table 10:** Random effects regressions: Of authors ranked between 51-100 per field.

	(1) MicroLow	(2) MacroLow	(3) EconometricsLow	(4) GameTheoryLow	(5) LaborLow	(6) ExperimentalLow
*Gender*	0.0587**	-0.0875**	-0.0148	-0.0647	-0.144**	-0.0222
	(0.0233)	(0.0375)	(0.0459)	(0.0470)	(0.0581)	(0.0310)
*Cohort 1960–1979*	-0.0159	-0.0742	0.0302*	0.0955***	0.101***	
	(0.0205)	(0.153)	(0.0170)	(0.0176)	(0.0355)	
*Cohort 1980–1999*	-0.00749	-0.0295	0.0526***	0.0909***	0.104***	0.134***
	(0.0227)	(0.154)	(0.0135)	(0.0155)	(0.0233)	(0.0403)
*Cohort ≥ 2000*	-0.0348			0.116		0.164***
	(0.0304)			(0.0740)		(0.0501)
*PhD Rank*	-0.000153	-0.000416***	-0.0000798	-0.000168	0.0000111	-0.000152*
	(0.000246)	(0.000134)	(0.0000960)	(0.000137)	(0.000182)	(0.0000849)
*Co-authors*	0.0144	-0.0122	0.00895	0.00686	-0.000967	0.00309
	(0.0163)	(0.0207)	(0.00682)	(0.00970)	(0.0125)	(0.00874)
*Trend*	-0.000484	0.00255	0.00302**	0.00423*	0.00522*	-0.00109
	(0.00314)	(0.00317)	(0.00150)	(0.00235)	(0.00305)	(0.00317)
*Initial Placement*	0.000150	-0.000176*	-0.0000409	-0.0000955	-0.000388***	-0.000249***
	(0.000152)	(0.0000986)	(0.0000650)	(0.000111)	(0.0000917)	(0.0000805)
*Initial Placement*	0.0000173	0.00000999	0.0000596	-0.0000575	-0.000118	-0.0000450
	(0.000136)	(0.000183)	(0.000146)	(0.000111)	(0.000106)	(0.000146)
Observations	480	465	488	489	479	500
*R* ^2^	0.05	0.17	0.05	0.08	0.31	0.15

Clustering standard errors on an author level. Standard errors in parentheses.

*p < 0.10,

**p < 0.05,

***p < 0.01.

Gender is a dummy variable that takes a value of one for male authors; We include cohort dummies to control for non-linear cohort-effects. Benchmark is the cohort < *1960*. PhD rank is the ranking of an author’s Alma mater. Co-authors contains the number of authors per paper. Trend denotes the paper’s number in the author’s publication history: 1 means first, 3 means third, and so on. Initial Placement captures the ranking of the university of the author’s initial placement. *R*^2^’s are overall *R*^2^.

Towards the top we observe that gender has heterogeneous effects, conditional on field. Male macroeconomists, econometricians and labor economists tend to publish in higher ranked journals whereas female game theorists tend to publish in better ranked journals among leading researchers, early in their careers. However, the latter results have to be considered with a grain of salt, given the moderate variation in gender composition within each field.

If we look at the authors ranked below the median, we observe that the relationship between an author’s Alma mater and his early career publication success is much weaker and not significant at a 10% level in four out of six fields. Put differently among increasingly skilled and potentially more homogeneous authors, the Alma mater signal seems to be of particular importance for early career success. Another interesting feature is the non-linear relationship between gender and early-career-success in macroeconomics and labor economics. Among lower ranked authors, female macro- and labor economists tend to be more successful in publishing in higher ranked journals, a relationship which reverts towards the top.

Second, we want to show that even if there is still some variation in ability present among our top researchers, that controlling for these differences does not affect our results. For that purpose we would like to include the current ranking of authors into our regressions as an imperfect proxy for marginal differences in ability levels. Clearly, there are some problems associated with including this particular variable since early career success might affect the current ranking at least indirectly. However, as we will show below, this variable has little impact on any of our results and the more homogeneous the author-pool (in terms of subsets) the less significant is this particular variable. This cannot be done with a random effects model, since we use the current ranking as a panel-identifier. Thus, we run OLS regressions with sequentiality dummies on the right hand side (our time-panel variable) and controlled for the current IDEAS ranking.

Table 11 shows our estimation results, focusing first on top 50 authors. We observe that the current ranking is not important to predict early career success in five out of six fields. Moreover, independently of the significance or insignificance of the current ranking, our main variable of interest—the ranking of an author’s Alma mater—is not affected by the inclusion of the current ranking.

**Table 11:** OLS regressions: Top 50 authors per field.

	(1) MicroTop	(2) MacroTop	(3) EconometricsTop	(4) GameTheoryTop	(5) LaborTop	(6) ExperimentalTop
*Gender*		0.140***	0.0867***	-0.0799	0.0994***	-0.0329
		(0.0384)	(0.0276)	(0.0612)	(0.0335)	(0.0349)
*Cohort 1960–1979*	-0.0446	-0.0738	-0.0262	-0.0308	-0.131	0.0118
	(0.0363)	(0.0907)	(0.0522)	(0.0510)	(0.103)	(0.0570)
*Cohort 1980–1999*	-0.0312	-0.112	-0.0324	-0.0423	-0.137	0.00347
	(0.0370)	(0.0924)	(0.0536)	(0.0511)	(0.102)	(0.0568)
*Cohort ≥ 2000*	-0.0697	-0.0253	-0.0451	-0.118**	0.0212	0.230*
	(0.0549)	(0.112)	(0.0699)	(0.0526)	(0.143)	(0.121)
*PhD Rank*	-0.000248**	-0.00174***	-0.000703***	0.00000463	-0.000740***	-0.000361***
	(0.000119)	(0.000503)	(0.000136)	(0.0000878)	(0.000186)	(0.0000912)
*Co-authors*	0.00959	0.00535	-0.0250**	0.0235**	0.0261	-0.00366
	(0.0116)	(0.0187)	(0.0105)	(0.0117)	(0.0160)	(0.0103)
*Initial Placement*	0.00000294	0.0000152	0.00000680	-0.0000494	-0.0000944	-0.0000591
	(0.000115)	(0.000115)	(0.000138)	(0.0000799)	(0.0000917)	(0.0000917)
*Ranking Now*	-0.000545	-0.000395	0.000910	-0.000859	-0.00183**	-0.000625
	(0.000610)	(0.000833)	(0.000605)	(0.000549)	(0.000880)	(0.000634)
Period Dummies	Yes	Yes	Yes	Yes	Yes	Yes
Observations	480	499	500	489	499	500
*R* ^2^	0.054	0.041	0.075	0.035	0.066	0.052

Clustering standard errors on an author level. Standard errors in parentheses.

*p < 0.10,

**p < 0.05,

***p < 0.01.

Gender is a dummy variable that takes a value of one for male authors; We include cohort dummies to control for non-linear cohort-effects. Benchmark is the cohort < *1960*. PhD rank is the ranking of an author’s Alma mater. Co-authors contains the number of authors per paper. Trend denotes the paper’s number in the author’s publication history: 1 means first, 3 means third, and so on—Dummies for each period used; Ranking Now is the current ranking of an author.

Table 12 shows our OLS results for the authors ranked below the median. Again, inclusion of the current ranking does not affect our results from above and the variable is not significant at a 10% level in any field. Table 13 considers all top-100 authors and includes the current ranking. Even here we observe that the current ranking only correlates with early career success in 3 out of six fields (econometrics, labor and experimental) and does not affect the influence of an author’s Alma mater. In all six fields we observe that, after controlling for current ranking, the ranking of the PhD granting university matters significantly at least at a 5% significance level. We also looked at predictors of average journal rankings in which our top authors published early in their careers, using simple OLS regressions. Since we could not control for sequentiality in this context, we again included our duration variable. We averaged over the number of co-authors per paper for each author. Our results reveal again that the only significant predictor for early career success is the ranking of an author’s Alma mater. This finding is significant at a 5% level for 5 out six fields and significant at a 10% level for the remaining field. We do not present these robustness checks for the sake of brevity.

**Table 12:** OLS regressions: Authors ranked from 51-100 per field.

	(1) MicroLow	(2) MacroLow	(3) EconometricsLow	(4) GameTheoryLow	(5) LaborLow	(6) ExperimentalLow
*Gender*	0.0594	-0.0960**	-0.0149	-0.0606	-0.143***	-0.0217
	(0.0516)	(0.0476)	(0.0301)	(0.0574)	(0.0499)	(0.0272)
*Cohort 1960–1979*	-0.0149	-0.0731	0.0300**	0.108***	0.101***	
	(0.0369)	(0.0759)	(0.0144)	(0.0178)	(0.0268)	
*Cohort 1980–1999*	-0.00683	-0.0218	0.0524***	0.105***	0.102***	0.0286
	(0.0352)	(0.0777)	(0.0123)	(0.0147)	(0.0183)	(0.0193)
*Cohort ≥ 2000*	-0.0337			0.136***		0.0610*
	(0.0628)			(0.0442)		(0.0346)
*PhD Rank*	-0.000152	-0.000365***	-0.0000805	-0.000155	0.00000246	-0.000161*
	(0.000187)	(0.0000981)	(0.0000657)	(0.000101)	(0.000121)	(0.0000876)
*Co-authors*	0.0162	-0.00811	0.00937	0.00438	0.00440	0.00536
	(0.0149)	(0.0164)	(0.00650)	(0.00835)	(0.0129)	(0.00889)
*Initial Placement*	0.000149	-0.000228**	-0.0000412	-0.0000842	-0.000384***	-0.000257***
	(0.000161)	(0.0000885)	(0.0000501)	(0.0000824)	(0.0000703)	(0.0000810)
*Ranking Now*	-0.0000638	-0.00128	-0.00000368	-0.000897*	-0.000814	-0.000805*
	(0.000777)	(0.000840)	(0.000300)	(0.000457)	(0.000639)	(0.000478)
Period Dummies	Yes	Yes	Yes	Yes	Yes	Yes
Observations	480	465	488	489	479	500
*R* ^2^	0.025	0.053	0.050	0.038	0.091	0.045

Clustering standard errors on an author level. Standard errors in parentheses.

*p < 0.10,

**p < 0.05,

***p < 0.01.

Gender is a dummy variable that takes a value of one for male authors; We include cohort dummies to control for non-linear cohort-effects. Benchmark is the cohort < *1960*. PhD rank is the ranking of an author’s Alma mater. Co-authors contains the number of authors per paper. Trend denotes the paper’s number in the author’s publication history: 1 means first, 3 means third, and so on—Dummies for each period used; Ranking Now is the current ranking of an author.

**Table 13:** OLS regressions: All top 100 authors.

	(1) Micro	(2) Macro	(3) Econometrics	(4) GameTheory	(5) Labor	(6) Experimental
*Gender*	0.0524	-0.0532	0.0296*	-0.0724*	-0.0814**	-0.0190
	(0.0351)	(0.0399)	(0.0172)	(0.0410)	(0.0391)	(0.0216)
*Cohort 1960–1979*	-0.0392	-0.0679	-0.00447	0.0302	-0.105	0.00404
	(0.0257)	(0.0584)	(0.0357)	(0.0296)	(0.0972)	(0.0563)
*Cohort 1980–1999*	-0.0293	-0.0654	0.00579	0.0217	-0.0946	0.0119
	(0.0259)	(0.0595)	(0.0357)	(0.0294)	(0.0973)	(0.0559)
*Cohort ≥ 2000*	-0.0697	-0.00682	0.00935	0.00506	-0.0998	0.0835
	(0.0433)	(0.0869)	(0.0554)	(0.0381)	(0.110)	(0.0657)
*PhD Rank*	-0.000228**	-0.000492***	-0.000209***	-0.0000789	-0.000334***	-0.000341***
	(0.000103)	(0.0000926)	(0.0000627)	(0.0000680)	(0.000104)	(0.0000645)
*Co-authors*	0.0110	0.000112	-0.00507	0.0134*	0.0120	0.000856
	(0.00927)	(0.0127)	(0.00578)	(0.00684)	(0.0103)	(0.00657)
*Initial Placement*	0.0000650	-0.000119*	-0.0000282	-0.0000760	-0.000223***	-0.000111*
	(0.0000960)	(0.0000668)	(0.0000564)	(0.0000571)	(0.0000586)	(0.0000624)
*Ranking Now*	0.00000873	-0.000344	-0.000328**	-0.000145	-0.000537**	-0.000595***
	(0.000240)	(0.000281)	(0.000133)	(0.000193)	(0.000272)	(0.000212)
Period Dummies	Yes	Yes	Yes	Yes	Yes	Yes
Observations	96	96	98	97	97	100
*R* ^2^	0.023	0.029	0.036	0.030	0.049	0.046

Clustering standard errors on an author level. Standard errors in parentheses.

*p < 0.10,

**p < 0.05,

***p < 0.01.

Gender is a dummy variable that takes a value of one for male authors; We include cohort dummies to control for non-linear cohort-effects. Benchmark is the cohort < *1960*. PhD rank is the ranking of an author’s Alma mater. Co-authors contains the number of authors per paper. Trend denotes the paper’s number in the author’s publication history: 1 means first, 3 means third, and so on—Dummies for each period used. Ranking Now measures the ranking of the author in the current top-100.

In summary, our results suggest that early career success among highly skilled researchers is strongly driven by the ranking of his or her Alma mater. Our robustness checks suggest that this effect is not an artifact of the estimation method or due to the clustering of certain observations into specific bins of the underlying distributions. Our results point towards the possible existence of gender effects, which may affect early career success heterogeneously, conditional on field. The number of co-authors does not seem to have a substantial and systematic effect on early career success among highly skilled authors. That being said, it might well have an effect on less able authors.

### The chances for top 5 publications

**Fig 6:**
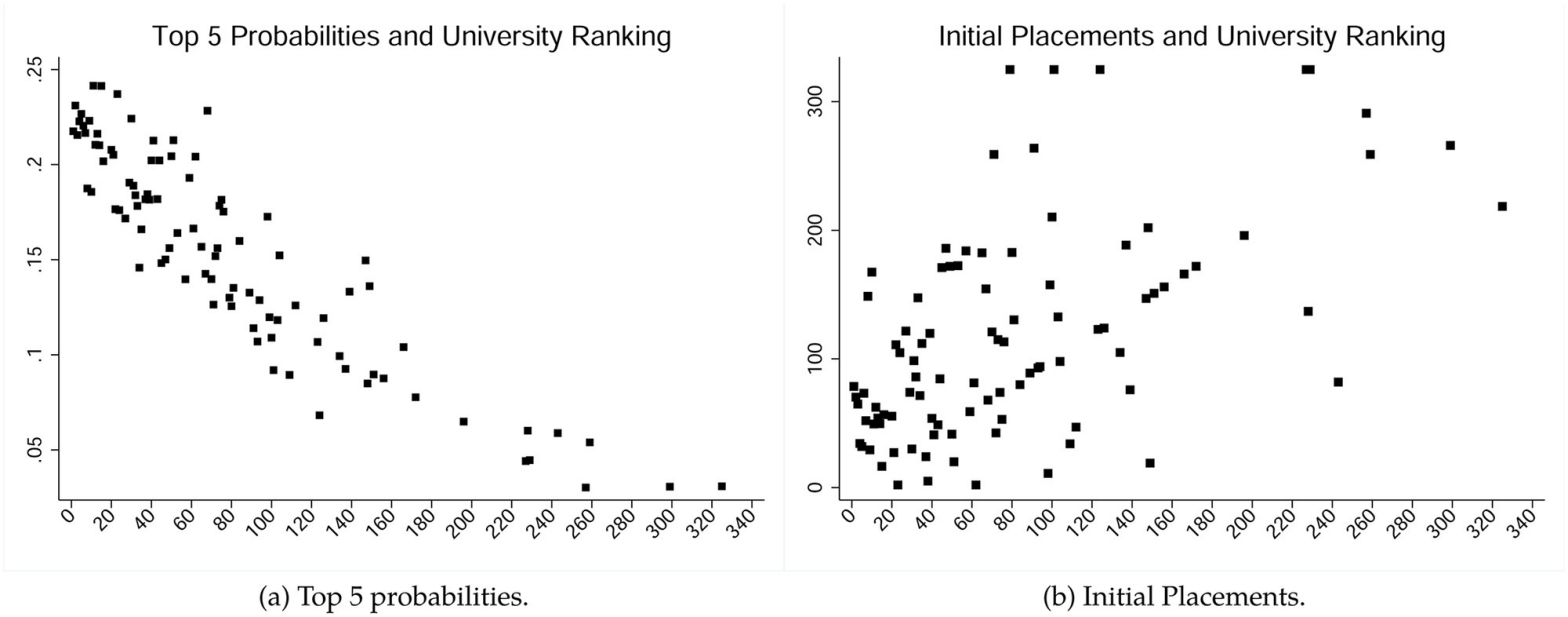
Average predicted probabilities for top 5 publications & average initial placement rankings, controlling for ability, as a function of Alma mater ranking. Panel (a) represents estimates for Top 5 probabilities as a function of Alma mater ranking; Panel (b) estimates initial placements as a function of Alma mater ranking.

In this subsection we explore predictors of publications in one of the top 5 journals in economics. We construct a dummy variable which takes the value of one if a paper was published either in the *Quarterly Journal of Economics, American Economic Review, Econometrica, Journal of Political Economy* or the *Review of Economic Studies* and is zero otherwise. Table 14 shows the estimates using a panel probit model. Aggregating over all fields shows again that the ranking of author’s PhD granting institution is an important predictor for publishing in a top 5 journal (column (1)). As a matter of fact, a reduction in the ranking of an author’s Alma mater by 100 ranks, reduces his probability to publish in one of these five journals by more than 30 percentage points. The relationship between Alma mater rankings and predicted average probabilities to publish in a top 5 outlet is illustrated in Fig 6.

**Table 14:** Panel probit models: Dummy variable which takes a value of 1 for a top 5 publication.

	(1) All	(2) Micro	(3) Macro	(4) Econometrics	(5) GameTheory	(6) Labor	(7) Experimental
*Gender*	-0.0489	0.759	-0.278	0.280	-0.336	0.00187	-0.163
	(0.132)	(0.609)	(0.313)	(0.401)	(0.344)	(0.229)	(0.251)
*Cohort 1960–1979*	-0.166	-0.508**	-0.162	0.279	0.318	0.257	-0.104
	(0.182)	(0.234)	(0.388)	(0.470)	(0.530)	(0.595)	(0.592)
*Cohort 1980–1999*	-0.302*	-0.422*	-0.327	0.185	0.0525	0.0895	-0.128
	(0.181)	(0.233)	(0.394)	(0.466)	(0.525)	(0.594)	(0.585)
*Cohort ≥ 2000*	-0.455*	-1.560***	-0.128	0.518	-0.595	-0.306	0.237
	(0.260)	(0.573)	(0.586)	(0.841)	(0.641)	(0.808)	(0.718)
*PhD Rank*	-0.00326***	-0.00152*	-0.00599***	-0.00216	-0.00147	-0.00308**	-0.00431***
	(0.000553)	(0.000841)	(0.00232)	(0.00136)	(0.00104)	(0.00131)	(0.00140)
*Co-Authors*	0.00929	0.0859	-0.0957	-0.0239	0.0622	0.0241	-0.00935
	(0.0323)	(0.0661)	(0.0811)	(0.0821)	(0.0684)	(0.0737)	(0.0747)
*Trend*	0.0237***	0.0102	0.0227	0.0381**	0.0304*	0.0222	0.0137
	(0.00767)	(0.0162)	(0.0165)	(0.0181)	(0.0176)	(0.0166)	(0.0199)
*Initial Placement*	-0.000761***	0.000161	-0.000630	-0.000686	-0.000700	-0.00179***	-0.00157
	(0.000310)	(0.000636)	(0.000551)	(0.000970)	(0.000795)	(0.000552)	(0.000959)
Observations	4889	930	934	958	948	959	950

Robust standard errors in parentheses. Standard errors in parentheses.

*p < 0.10,

**p < 0.05,

***p < 0.01.

Gender is a dummy variable that takes a value of one for male authors; We include cohort dummies to control for non-linear cohort-effects. Benchmark is the cohort < *1960*. PhD rank is the ranking of an author’s Alma mater. Co-authors contains the number of authors per paper. Trend denotes the paper’s number in the author’s publication history: 1 means first, 3 means third, and so on. Initial Placement captures the ranking of the university of the author’s initial placement.

**Fig 7:**
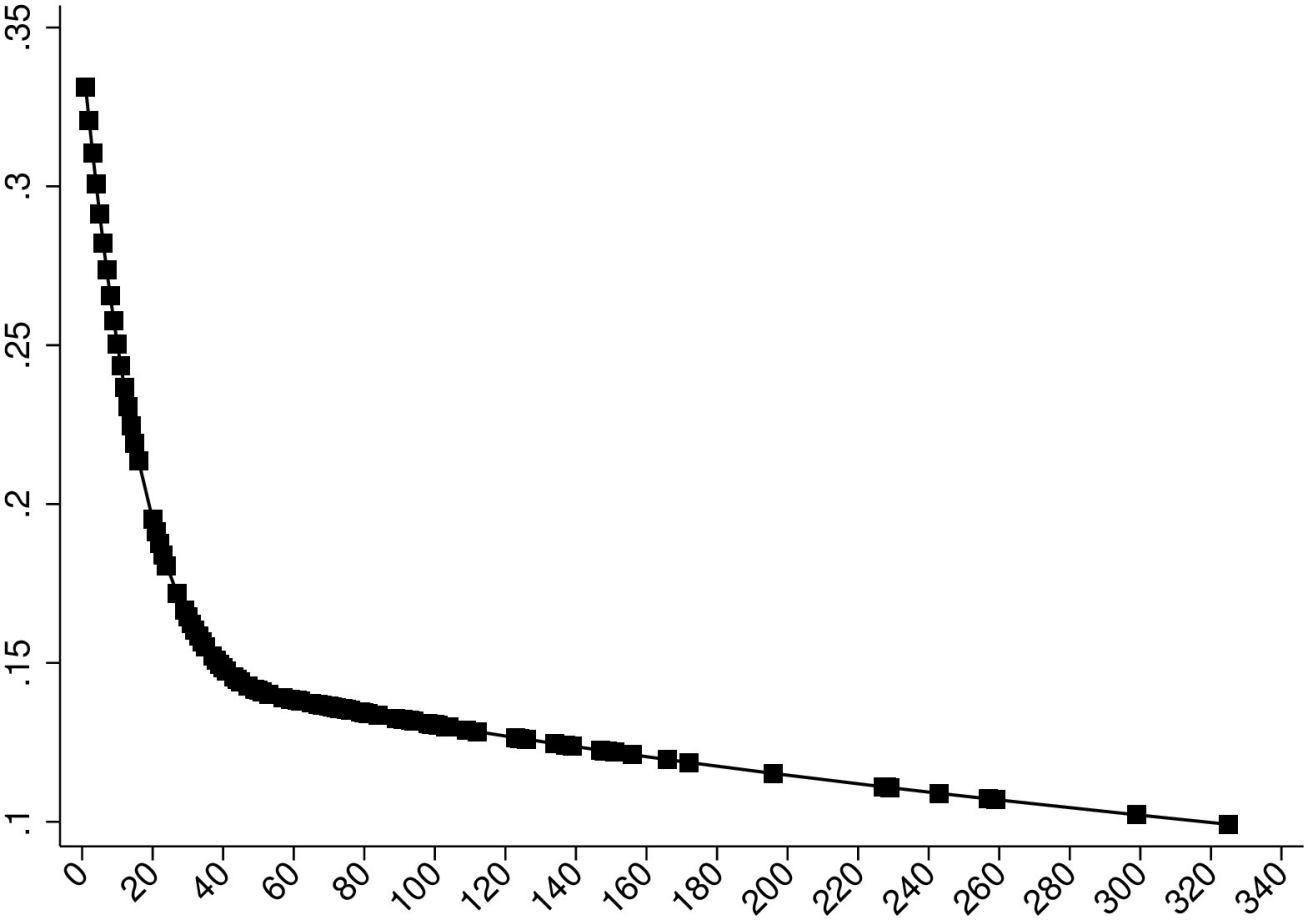
Predicted acceptance probabilities to a top 5 journal against Alma mater ranking at the time of graduation using splines.

We also extend our probit models, allowing for changes in the coefficients associated with the rankings of PhD granting institutions using cubic splines. Our previous results barely change with an estimated probability that a paper ends up in a top 5 journal being 30% (top 10 *Almae matres*; Standard deviation: 0.02), 23% (top 11-20; Standard deviation: 0.01), 17.4% (top 21-30; Standard deviation: 0.01), 15% (top 31-50; Standard deviation: 0.006), 13.5% (top 51-100; Standard deviation: 0.002) and 10.2% (Below 100; Standard deviation: 0.004) respectively. Nevertheless, an interesting insight is that graduates from top 10 *Almae matres* have a comparative advantage to publish in a top 5 journal of almost 13 percentage points over the colleagues who graduated from universities in the range of 21 to 30. Fig 7 shows the relationship between Alma mater rankings and predicted probabilities to publish in the top 5 using cubic splines. We also controlled for initial job placement-rankings (both linearly and via cubic splines), which render insignificant in all our top 5 regressions and do not affect the sign or the significance of the Alma mater effect. We also controlled for current ranking of authors, in case “ability” is not fairly homogeneous and affects early career success in terms of publishing in a top 5 journal. Again, we do not observe in any of our regressions that the current ranking matters (i.e.: the coefficient associated with the ranking is insignificant and has no effect on the sign or the significance of the Alma mater effect).

Our results in Table 14 further suggest field specific differences. Prestige of *Almae matres* plays an important role to publish in a top 5 journal among fairly homogeneous and highly skilled authors in macro-, labor- and experimental economics (1% and 5% significance), a mild role in microeconomics (10% significance) and no role in game theory and econometrics.

We also observe mild cohort-effects when we look at predictors for top publications. Indeed, without controlling for field specific differences, publishing in very prestigious outlets has become significantly more difficult over time. This seems particularly true for microeconomists. We also extended our controls by accumulated top 5 publications for each author to see whether the sum of top 5 publications until the *n*^*th*^ − 1 publication affects the probability that the *n*^*th*^ publication also ends up in a top 5 outlet. Within the set of the first 10 publications, we observe that this variable is neither significant from an aggregate nor a field perspective and has no effects on the signs or significances of our other variables. Hence, again for the sake of brevity, we omit reporting these separate probit regressions.

We also looked into the Markov transition probabilities of getting a top 5 publication at some point in our author’s careers, conditional on having and not having a most recent top 5 publication. Our results indicate that the probability of getting a top 5 publication right after a non-top 5 publication is about 18%. This transition probability increases in our author pool to about 33%, if the most recent publication of an author was another top 5 publication. There is very little and no significant difference with respect to this effect across fields.

Next, we construct a dummy variables, *QJE (JPE)*, which take values of one if a paper was published in the *Quarterly Journal of Economics* (QJE hereafter) or the *Journal of Political Economy* (JPE hereafter). The former journal is edited by Oxford University Press on behalf of the economics department of Harvard, the latter is edited by Chicago University Press. We observe that out of the 158 QJE publications in our data-set, 38 were written by Harvard graduates, 44 by MIT graduates and only 6 by graduates of the University of Chicago. Hence about 52% of the papers, published in the QJE, were written by graduates from the greater “Boston area”, whereas less than 4% were written by Chicago graduates. Our data also reveal that out of 168 JPE publications, 19 (27) were written by Harvard (MIT) graduates, whereas 19 were written by Chicago graduates. For the other top 5 journals we have the following patterns: American Economic Review: Out of 299, 43 (77) by Harvard (MIT) and 13 by Chicago graduates (40% and 4%), Econometrica: Out of 379, 13 (45) by Harvard (MIT) and 2 by Chicago graduates (15% and 0.5%), Review of Economic Studies: Out of 189, 12 (37) by Harvard (MIT) and 8 by Chicago graduates (26% and 4%).

To test whether we observe “ZIP code” or “in-house-favoritism” effects, we construct a dummy variable which takes a value of one if a paper was published in the QJE (QJE dummy) and another dummy variable if a paper was published in the JPE (JPE dummy). Next, we construct dummies for Harvard, MIT and Chicago *Almae matres*. We run random effects regressions, to check for the partial effects of these variables on the QJE/JPE dummies in two separate regressions. The aggregate and field-specific results for QJE publications are shown in Table 15. Table 16 shows aggregate and field-specific results for JPE publications.

**Table 15:** Random effects models: Dummy variable which takes a value of 1 for a QJE publication.

	(1) All	(2) Micro	(3) Macro	(4) Econometrics	(5) GameTheory	(6) Labor	(7) Experimental
*Gender*	-0.0161	0.0297	-0.0190	0.00480	-0.0416**	-0.0621**	0.0239
	(0.0147)	(0.0470)	(0.0378)	(0.0119)	(0.0166)	(0.0252)	(0.0185)
*Cohort 1960–1979*	-0.0139	0.0228	-0.0806*	-0.0341**	0.0127	0.0153	0.0430
	(0.0152)	(0.0261)	(0.0483)	(0.0138)	(0.0242)	(0.0721)	(0.0449)
*Cohort 1980–1999*	-0.00320	0.0360	-0.0793	-0.0284**	0.0132	0.0347	0.0589
	(0.0150)	(0.0263)	(0.0493)	(0.0136)	(0.0240)	(0.0716)	(0.0446)
*Cohort ≥ 2000*	0.00686	0.0498	-0.0168	-0.0354	0.0224	0.0654	0.0369
	(0.0200)	(0.0441)	(0.0738)	(0.0268)	(0.0271)	(0.0855)	(0.0523)
*Trend*	0.00154*	0.00421**	0.00164	0.000628	0.000663	0.00213	-0.000700
	(0.000817)	(0.00194)	(0.00249)	(0.000810)	(0.00117)	(0.00232)	(0.00179)
*PhD Rank*	-0.0000808**	-0.0000717	-0.000218	-0.0000245	0.0000410	-0.0000209	-0.000120
	(0.0000322)	(0.0000891)	(0.000177)	(0.0000419)	(0.0000440)	(0.000119)	(0.0000791)
*Co-authors*	0.00215	-0.00142	0.00332	-0.00369	0.00291	0.00620	0.00520
	(0.00296)	(0.00790)	(0.0116)	(0.00335)	(0.00414)	(0.00918)	(0.00626)
*Harvard*	0.0653***	-0.00945	-0.0108	-0.00211	-0.0110	0.0995***	0.164***
	(0.0187)	(0.0244)	(0.0327)	(0.0136)	(0.0234)	(0.0216)	(0.0253)
*Chicago*	0.00153	0.0624*	-0.0652*	-0.00000647	-0.0130	-0.0113	-0.0268
	(0.0142)	(0.0273)	(0.0385)	(0.0232)	(0.0235)	(0.0313)	(0.0606)
*MIT*	0.0504***	0.0293	0.0434**	0.0280***	0.00559	0.0384*	0.0502**
	(0.00789)	(0.0186)	(0.0201)	(0.00986)	(0.0127)	(0.0201)	(0.0248)
*Initial Placement*	-0.0000178	0.0000592	0.00000417	0.00000838	-0.0000798**	-0.000184***	0.0000132
	(0.0000234)	(0.0000691)	(0.0000655)	(0.0000293)	(0.0000350)	(0.0000571)	(0.0000620)
Observations	4889	930	934	958	948	959	950
*R* ^2^	0.15	0.14	0.15	0.20	0.14	0.41	0.40

Robust standard errors in parentheses. Standard errors in parentheses.

*p < 0.10,

**p < 0.05,

***p < 0.01.

Gender is a dummy variable that takes a value of one for male authors; We include cohort dummies to control for non-linear cohort-effects. Benchmark is the cohort < *1960*. PhD rank is the ranking of an author’s Alma mater. Co-authors contains the number of authors per paper. Trend denotes the paper’s number in the author’s publication history: 1 means first, 3 means third, and so on. Initial Placement captures the ranking of the university of the author’s initial placement. Harvard, MIT and Chicago are Alma mater dummies for those three schools. *R*^2^’s are overall *R*^2^.

**Table 16:** Random effects models: Dummy variable which takes a value of 1 for a JPE publication.

	(1) All	(2) Micro	(3) Macro	(4) Econometrics	(5) GameTheory	(6) Labor	(7) Experimental
*Gender*	0.0147*	0.0315	-0.0174	0.0205	0.0153	0.0562**	0.00901
	(0.00859)	(0.0529)	(0.0411)	(0.0244)	(0.0215)	(0.0256)	(0.0133)
*Cohort 1960–1979*	-0.0638**	-0.158***	-0.0129	0.0258	-0.0238	0.0805	-0.0632**
	(0.0302)	(0.0294)	(0.0525)	(0.0284)	(0.0313)	(0.0728)	(0.0322)
*Cohort 1980–1999*	-0.0682**	-0.129***	-0.0112	0.00873	-0.0387	0.0811	-0.0808**
	(0.0299)	(0.0296)	(0.0537)	(0.0280)	(0.0310)	(0.0723)	(0.0320)
*Cohort ≥ 2000*	-0.0766**	-0.164***	-0.0485	-0.00698	-0.0446	0.0608	-0.0664*
	(0.0310)	(0.0497)	(0.0803)	(0.0551)	(0.0350)	(0.0862)	(0.0376)
*Trend*	-0.000138	-0.00402*	0.00253	-0.000732	0.000476	0.00294	-0.000576
	(0.000824)	(0.00236)	(0.00279)	(0.00147)	(0.00137)	(0.00227)	(0.00144)
*PhD Rank*	-0.0000619*	0.000112	-0.000299	-0.000111	0.0000238	-0.0000287	-0.0000149
	(0.0000379)	(0.000100)	(0.000192)	(0.0000860)	(0.0000569)	(0.000121)	(0.0000567)
*Co-authors*	-0.000864	0.00609	-0.00566	-0.00573	-0.00246	-0.00256	0.00270
	(0.00320)	(0.00941)	(0.0129)	(0.00627)	(0.00497)	(0.00908)	(0.00494)
*Harvard*	0.00987	-0.00241	0.00834	0.00511	-0.0226	0.0195	0.0144
	(0.0137)	(0.0274)	(0.0356)	(0.0279)	(0.0302)	(0.0219)	(0.0182)
*Chicago*	0.0500***	0.0972***	0.00240	-0.0333	-0.0155	0.0661**	-0.0154
	(0.0228)	(0.0308)	(0.0419)	(0.0476)	(0.0304)	(0.0317)	(0.0435)
*MIT*	0.0119	0.0203	-0.0365*	0.0134	-0.00694	0.0309	0.0267
	(0.00963)	(0.0209)	(0.0219)	(0.0203)	(0.0164)	(0.0204)	(0.0178)
*Initial Placement*	-0.0000585**	-0.000160**	-0.0000856	0.00000949	-0.0000553	-0.000179***	-0.0000685
	(0.0000229)	(0.0000778)	(0.0000713)	(0.0000603)	(0.0000452)	(0.0000579)	(0.0000445)
Observations	4889	930	934	958	948	959	950
*R* ^2^	0.1 0	0.43	0.08	0.08	0.09	0.22	0.19

Robust standard errors in parentheses. Standard errors in parentheses.

*p < 0.10,

**p < 0.05,

***p < 0.01.

Gender is a dummy variable that takes a value of one for male authors; We include cohort dummies to control for non-linear cohort-effects. Benchmark is the cohort < *1960*. PhD rank is the ranking of an author’s Alma mater. Co-authors contains the number of authors per paper. Trend denotes the paper’s number in the author’s publication history: 1 means first, 3 means third, and so on. Initial Placement captures the ranking of the university of the author’s initial placement. Harvard, MIT and Chicago are Alma mater dummies for those three schools. *R*^2^’s are overall *R*^2^.

First, we observe that the ranking of *Almae matres* still matters for publishing in the QJE (column (1)) and that the rankings of initial placements do not matter significantly. Graduating from Harvard or the MIT does indeed affect the success of publishing in the QJE, whereas graduating from Chicago does not seem to have a statistically significant effect –after controlling for the ranking of *Almae matres*. We observe field specific differences. Harvard graduates, working on labor- or experimental economics, seem to have a particular advantage over their peers, whereas MIT graduates seem to have an additional advantage in macroeconomics and econometrics to publish in the QJE. Using panel probit regressions, we are able to quantify the relative advantage of Harvard and MIT graduates via marginal effects. We omit the presentation of these regressions for the sake of brevity since the results from the random effects models do not change qualitatively. The results indicate that the relative advantage of Harvard (MIT) graduates amounts to 5 (4) percentage points, which is significant at a 1% (5%) level.

Table 16 shows aggregate and field-specific results for JPE publications. We observe that the ranking of *Almae matres* matters only at a 10% significance level and that graduating from Harvard or the MIT has no statistically significant effect on publishing in the JPE. However, graduating from Chicago affects publication chances at the JPE significantly at a 5% level. The field specific regressions reveal that the aggregate effect seems to be generated by micro- and labor-economics. We again compute the marginal effects of a Chicago degree to publish in the JPE, using panel probit regressions. The marginal effect amounts to a significant increase of 3.7 percentage points in acceptance probability. We also observe strong cohort effects, suggesting that it has become increasingly difficult to publish in the JPE over time. We also added to both sets of regressions (QJE and JPE) dummy variables for the remaining top 10 institutions around the time of graduation of most of our authors (ordered based on ranking): Harvard, Chicago, MIT, Berkeley, Princeton, Stanford, Northwestern, U Penn, Yale and NYU. For our JPE regressions *only* Chicago remains a significant predictor and no other dummy becomes significant. For our QJE regressions, Harvard and MIT remain significant and only (if anything) the Princeton dummy becomes significant at a 10% level. No other school dummy becomes significant at a 10% significance level. We also rerun the same regressions with Econometrica, American Economic Review (AER) and Review of Economic Studies (ReStud) dummies on the left hand side. In our Econometrica regressions only the Princeton and Stanford dummies are significant. In our ReStud regressions we observe that only the Princeton and MIT dummies are significant at a 5% level. In our AER regressions 5 out of the 10 Alma mater dummies are significant at a 5% level (Berkeley, U Penn and NYU are insignificant; Chicago and Yale are only significant at 10% level). In other words, there is no systematic pattern suggesting that it is always the same set of schools that has an advantage over all other schools across all top 5 journals. E.g.: Within this set of 10 schools the top 3 universities, which generated the largest number of graduates who received Nobel prizes, were Harvard followed by Chicago and MIT. This additional robustness check makes the evidence on QJE- and JPE-publications more striking since both journals can be assigned easily to specific economics departments. Moreover, graduates from these departments seem to be held in high regard by the corresponding editors.

### Initial placements

Our data also enables us to look into predictors of initial placements and whether *Almae matres* affect initial placements significantly, conditional on field, among authors who all turned out to be highly skilled. Indeed, if the placement market worked efficiently and only considered skill, innate ability and maybe effort, there should be no significant correlation between the initial placements of our authors and the rankings of their Alma mater.

First, we run simple quantile regressions using the rankings of initial placements as endogenous variable, controlling for fields via dummy variables. The results are shown in the first column of Table 17, using the field of microeconomics as a benchmark. Column 2 of the same Table focuses only on placements in universities.

**Table 17:** Quantile regression models: Ranking of university or other institution.

	(1) Q1	(2) Q2Academia	(3) Q3	(4) Q4	(5) Q5Academia
*Macro*	11.00	1.000	4.432	-9.703	-7.243
	(11.99)	(4.543)	(3.574)	(8.338)	(5.090)
*Econometrics*	15.00	15.00	0.342	-14.70	-5.266
	(12.08)	(11.63)	(2.964)	(11.43)	(7.547)
*Game Theory*	19.00*	17.00***	2.814	-12.72	-1.347
	(9.697)	(5.563)	(5.568)	(10.53)	(7.195)
*Labor*	19.00	9.000	9.167**	-6.351	-4.120
	(18.52)	(9.170)	(4.263)	(11.46)	(3.240)
*Experimental*	51.00***	41.00***	13.64**	2.541	18.28
	(9.517)	(7.555)	(6.741)	(15.24)	(13.74)
*PhD Rank*			0.931***		
			(0.0332)		
*Gender*			-0.00833	2.207	3.407
			(21.13)	(20.05)	(2.865)
*PhD Year*			0.214	0.211	0.0970
			(0.149)	(0.159)	(0.0828)
*MacroRank*				0.928***	0.877***
				(0.0533)	(0.101)
*EconometricsRank*				0.943***	0.841***
				(0.106)	(0.317)
*GameRank*				0.964***	0.664***
				(0.0318)	(0.214)
*LaborRank*				0.955***	0.923***
				(0.0838)	(0.0482)
*ExperimentalRank*				0.852***	0.485*
				(0.112)	(0.271)
Observations	518	472	518	511	472
*Pseudo* − *R*^2^	0.02	0.04	0.20	0.17	0.18

Bootstrapped standard errors in parentheses. Standard errors in parentheses.

*p < 0.10,

**p < 0.05,

***p < 0.01.

Gender is a dummy variable that takes a value of one for male authors; PhD Year controls for graduation years. PhD rank is the ranking of an author’s Alma mater.

As expected, we observe that experimental economists place worst, followed by game theorists. Among our authors, the placements in other fields do not differ significantly. Column 3 extends our set of covariates, including also the ranking of *Almae matres*. We observe that the field-specific placement differences either decrease quite substantially or are not significant anymore. Only in the case of labor economics we observe an emerging field-specific difference, after controlling for the ranking of an author’s PhD granting institution.

Our results indicate that the coefficient associated with the ranking of an author’s Alma mater is not significantly different from one, suggesting that an increase in the ranking of a PhD granting institution by one translates into an equally sized impact on initial placement success. This is surprising, given that all our authors turned out to be extremely skilled and influenced their relative fields significantly from an ex-post perspective. Fig 6 shows the relationship between initial placements and the rankings of *Almae matres* for our authors.

In column 4 we interact the field dummies with the rankings of *Almae matres* and in column 5 we focus only on university placements. We observe that the prestige of *Almae matres* for university placements among highly skilled researchers is most important and strongest in the fields of labor economics, macroeconomics and econometrics. An F-test suggests that it is significantly less important in game theory (5% significance) and experimental economics (1% significance). None of the other covariates seem to influence the initial job placement success of our authors. We also used OLS regressions and find essentially the same results, looking at conditional averages instead of conditional medians.

In summary, we observe that the rankings of *Almae matres* have a substantial influence on the success of initial job placements. However, one might argue that –in contrast to the findings on publications– this result is slightly less “troubling”. After all, universities employ researchers not only based on academic skill but also based on other abilities (e.g.: networking, social skills, teaching etc.) which might correlate with being at certain prestigious *Almae matres*. The same argument does not hold, in our opinion, when it comes to scientific publications, which supposedly contribute to a greater good and should therefore not depend on anything else but skill, ability and quality. While our results do not necessarily suggest that authors from higher ranked *Almae matres* face an advantage, they reveal that lower ranked newcomers are relatively discounted simply based on the ranking of their PhD granting universities.

## Discussion

We estimate the effects of affiliations on a researcher’s chances to publish his work in economics journals early in his career. Since the ability of a researcher is not publicly known at the time of graduation, any information about the researcher’s ability might be useful information for editors and referees. In this section, we discuss potential explanations of the significance of the Alma mater effect we observe.

### Ability

The quality of original research is inherently hard to measure or quantify—the value comes from novelty, which is visible only in the long run. Therefore, editors and other decision makers infer ability from everything they can observe. In our regressions, we had no explicit control to proxy ability, and the natural reaction to our estimates is to notice that our results could be driven by this unobservable: more able authors are likely to be more able in their youth, and therefore are more likely to be admitted to higher-ranked PhD programs. While this argument is plausible, we observe that the Alma mater effect remains significant for the 10th paper submitted, even controlling for past publication history and the placement information. Also, the authors we’ve chosen for our sample are the best researchers in their fields, and their abilities are likely to be high enough to not be an issue for publication. Moreover, the current ranking of these researchers is not a significant variable in our regressions or has no effect on the significance of the Alma mater effect, further underlining that the effect of an increase in ability in our very skilled author pool is not high.

### Nurture

Even ignoring the informative content of affiliation, having it statistically significant does not necessarily imply any inefficiency. After all, studying in a highly-ranked PhD program can teach soft skills, such as paper formatting or science-vocabulary, that are not available to graduates of less ranked schools. While this is a plausible explanation, it remains unclear why the benefit of these soft skills is so persistent that it remains to be informative after the 5th publication. Moreover, our interaction terms in our pooled regressions suggest that the Alma mater effect does not seem to vanish over time, within the set of first ten publications. Same can be said about the nurturing effects of getting placed at highly-ranked places: if there was a strong nurturing effect, it should not be that much weaker than the Alma mater effect, since most publications come out after the placement.

### Personal relations

There is some benefit of having a personal connection to editors: graduates of Harvard University and of University of Chicago have a significantly higher chance of getting a top-5 publication if it is either in the QJE or JPE, even after controlling for individual fixed effects. This might be a voluntary decision of the graduates to publish their *chef d’œuvre* in their Alma mater’s journal when one, arguably, would be accepted to any other journal. However, it might also be evidence on editorial favoritism, which has been documented before [3–6] or on “like-mindedness” [22]: Editors from some highly ranked university are more likely to share the views of graduates from the same institution. Like-mindedness, as a determinant of editorial decision making, is consistent with the evidence in [23], suggesting that “agreement” *within* economic departments is higher than *across* economic departments. [22] argue that like-mindedness goes against scientific innovation, diversity and sometimes maybe even progress. Like-mindedness as a determinant for our Alma mater and ZIP-code effects is also in line with the evidence on citation-rings presented in [24].

### Advisor editor

Even the most impartial cannot ignore their subconscious. If an advisor of a graduate is an editor of a journal where the said graduate has submitted a paper to, the advisor’s duties dictate to teach the graduate what to do to maximize the probabilities of acceptance, and the editor’s duties dictate to share the information about the brilliance of the advisee with other decision makers. It would be interesting to control for whether the advisor of the researcher in our dataset is the editor of the journal where the paper ended up. Not having this control can lead to the significance of the Alma mater variable because better rated PhD programs tend to have more editors among their faculty [22]. However, the same argument applies if the editor is not a direct advisor, but a placement director, or a committee member, just had a coffee with the said graduate, attended a workshop presentation etc. Obviously, it would be hard to take into account publication lags and not knowing whether the advisor was actually the editor for that specific paper. We do not have this particular control in our study, but that explanation would make the most difference among top-20 to 30, not among top-10 to 20, as our estimates show.

### Co-authors

Closely intertwined with a few of the previous explanations is the story about co-authors: arguably, co-authors appear from the author’s neighborhood, which includes both the graduating program and the first placement. Arguably, at better ranked departments, one should expect co-authors of higher ability. Except for experimental economics, the average number of co-authors is 0.6 in our dataset, which translates to 50% of solo papers (experimental has 35%). Moreover, keeping only solo papers in the dataset retains the Alma mater effect.

## Conclusion

The prestige of *Almae matres* has a substantial and significant effect on the early publication success of economists, even after controlling for ex-post revealed ability levels. Among highly proficient researchers, economists from lower ranked *Almae matres* tend to be discounted significantly across all fields, suggesting a potential inefficiency in the dissemination process of scientific thought. The Alma mater effect is large in size: if a top-10 graduate has a 30% chance of publishing in a top-5 journal, graduating instead from a top-30 institution lowers his chances to 17%, and lowering his Alma mater ranking further to 100+ lowers his chances to 10%. This sizable effect is surprising since all of our top authors influenced their respective fields along various dimensions in the long run. Our results further suggest that “in-house” journals favor “in-house” graduates in addition to overall Alma mater effects. Furthermore, the quantity of co-authors has negligible effects on publication success except, maybe, in the field of game theory. The effect of gender on early career success is ambiguous and tends to be field-specific, but female researchers are very underrepresented in the dataset that we study.
